# Cloning, Annotation and Developmental Expression of the Chicken Intestinal *MUC2* Gene

**DOI:** 10.1371/journal.pone.0053781

**Published:** 2013-01-21

**Authors:** Zhengyu Jiang, Todd J. Applegate, Amy C. Lossie

**Affiliations:** 1 Department of Animal Sciences, Purdue University, West Lafayette, Indiana, United States of America; 2 Department of Medicine, Indiana University School of Medicine, Indianapolis, Indiana, United States of America; National Institute on Aging, United States of America

## Abstract

Intestinal mucin 2 (*MUC2*) encodes a heavily glycosylated, gel-forming mucin, which creates an important protective mucosal layer along the gastrointestinal tract in humans and other species. This first line of defense guards against attacks from microorganisms and is integral to the innate immune system. As a first step towards characterizing the innate immune response of MUC2 in different species, we report the cloning of a full-length, 11,359 bp chicken *MUC2* cDNA, and describe the genomic organization and functional annotation of this complex, 74.5 kb locus. *MUC2* contains 64 exons and demonstrates distinct spatiotemporal expression profiles throughout development in the gastrointestinal tract; expression increases with gestational age and from anterior to posterior along the gut. The chicken protein has a similar domain organization as the human orthologue, with a signal peptide and several von Willebrand domains in the N-terminus and the characteristic cystine knot at the C-terminus. The PTS domain of the chicken MUC2 protein spans ∼1600 amino acids and is interspersed with four CysD motifs. However, the PTS domain in the chicken diverges significantly from the human orthologue; although the chicken domain is shorter, the repetitive unit is 69 amino acids in length, which is three times longer than the human. The amino acid composition shows very little similarity to the human motif, which potentially contributes to differences in the innate immune response between species, as glycosylation across this rapidly evolving domain provides much of the musical barrier. Future studies of the function of MUC2 in the innate immune response system in chicken could provide an important model organism to increase our understanding of the biological significance of MUC2 in host defense and highlight the potential of the chicken for creating new immune-based therapies.

## Introduction

The vast majority of the gastrointestinal tract is covered by a mucosal surface, which creates an important biological barrier that shields the epithelial lining. The top layer of the mucus gel surface, which is the first line of the innate immune defense, is composed primarily of a family of proteins called mucins (MUC). Mucin family members are broadly grouped into secretory and membrane-associated mucins. Membrane associated mucins are involved in signal transduction, oncogenic processes and/or gel formation [1]. Secretory gel-forming mucins (i.e. MUC2, MUC5AC, MUC5B, MUC6, MUC7 and MUC19) contain at least one repetitive domain rich in Pro, Thr and Ser (i.e. the PTS-domain), as well as von Willebrand domains (B, D or C), a cysteine rich domain (CysD), and a cystine knot (CT) [2], [3]. O-linked glycosylation occurs in the PTS domain, while the VWB, VWD, VWC, CysD and CT regions facilitate oligomerization and polymerization. In the small and large intestine, the primary gel-forming mucin is *MUC2*, although there are detectable levels of *MUC5AC* and *MUC6* in the large intestine [4].

Human MUC2 is a large (5179 amino acid) heterologous glycoprotein that can be modified posttranslationally with more than 100 different oligosaccharides [5]. The oligosaccharides attach along the middle of the protein throughout the mucin domain [6]. The cystine knots at the C-terminus facilitate homodimerization in the endoplasmic reticulum [7], while trimerization occurs in the Golgi through the formation of disulfide bonds at the N-terminus [8]. This produces a 6-membered homopolymer that potentially oligomerizes into hexagonal sheets [4], [9], [10], [11]. Interactions between internal CysD sites create the 3D architecture of the mucosal gel surface [12]. In the intestinal lumen, the charged sugar chains efficiently trap water molecules, creating a stable continuous network that functions analogously to a protective semi-permeable membrane [13]. This protective structure is continually assaulted by physical shear stress due to luminal fluid flow, microbial forging and erosion from proteases or chemical degradation [4].

MUC2 is fundamental in maintaining the architecture of the gel layer on the intestinal surface and in preventing microorganisms from approaching the innermost mucus layer [6]. Alternate splicing of *MUC2* and the heterologous nature of the attachment of the sugar molecules generate a highly heterogeneous mucin gel layer, which creates a broad innate defense mechanism within the gastrointestinal tract. Deficiency of or missense mutations in *Muc2* causes the epithelial barrier to become permeable to bacteria, leading to colonic inflammation and spontaneous colitis in mice [14], [15], as well as increased susceptibility to infection by enteric nematodes [16]. In humans, rare short *MUC2* exonic minisatellites comprised of sequences from the tandem repeat PTS cassettes, have been associated with the onset of gastric cancer [17].

Functional annotation of *MUC2* in humans indicates the presence of two polymorphic PTS cassettes [18] and 11 alternatively spliced *MUC2* transcripts (UniProtKB, Swiss-Prot) [19]. In addition, analysis of *MUC2* in the LS174T derived HM7 colon cancer cell line led to the identification of a transcript variant that lacked the second PTS domain [20]. The presence of this highly polymorphic PTS VNTR (variable number of tandem repeats) has inhibited the resolution of the full-length mRNA, as well as the functional annotation of the complete DNA sequence in many species, including mouse and human [4], [9], [10]. Despite these efforts, the precise annotation of these alternatively spliced *MUC2* transcripts remains incomplete, and the length of the PTS domain, which is predicted to span 55–110 cassettes, remains highly polymorphic within the human population [18]. Although the biological relevance of these alternatively spliced products in human is not fully understood, it is believed that they are associated with pathogenesis of intestinal diseases. Although functional studies in mice have indicated that *Muc2* plays roles in the biology and health of the gut [15], [21], [22], [23], the function of the PTS domain in mice is less clear, due to the annotation of a relatively short and imprecise repetitive cassette [24].

Evolutionary studies predict that the gel forming mucins share a common ancestor with lower metazoa, as their domain structures are well conserved across a wide range of species from invertebrates to humans [3], [25]. However, relatively few *MUC* genes have been identified in avians and amphibians. The first *Mucin* gene cloned in chicken was ovomucin alpha-subunit [26], now annotated as *MUC5B*. *In silico* predictions [3], [27] and annotation of short mRNAs and expressed sequence tags (ESTs) have generated a putative partial *MUC2* cDNA in chicken. However, these studies have provided very little functional annotation evidence of the genomic organization of the chicken *MUC2* locus. To determine the structure, expression, biosynthesis and gene signatures of intestinal mucins from a functional and evolutionary perspective, we cloned the chicken *MUC2* cDNA that encodes the MUC2 peptide backbone. We achieved this by analyzing and assembling more than 85 cDNA clones that were generated by overlapping RT-PCR products, rapid amplification of cDNA ends (RACE), sequencing of ESTs, and incorporating functional annotation data (i.e. mRNAs and ESTs) from the UCSC database [28] and NCBI [29]. We also compared our sequence to the predicted chicken cDNA (http://www.medkem.gu.se/mucinbiology/databases/). We found that the 11,359 bp chicken cDNA spans 74.5 kb of genomic DNA and is comprised of at least 64 exons. *MUC2* is expressed in multiple regions of the gastrointestinal tract, and we detected transcripts as early as embryonic day 14.5. We found several alternatively spliced products, and characterized the splice junctions of one of these transcripts. We determined that the chicken MUC2 protein is remarkably similar to human and mouse outside of the central PTS domain, but is highly divergent within this central repetitive structure. In humans, this PTS domain is highly glycosylated by *O*-glycans in the Golgi, and it is predicted that these posttranslational modifications largely contribute to the innate immune response, as proteolytic cleavage of these sugar chains occurs in the outer mucus layer when these molecules come into contact with foreign pathogens [30]. It will be interesting to compare the posttranslational modifications in chicken with other species, especially given the high degree of divergence in this region.

## Methods

### Tissue biopsy, total RNA isolation

Ethics statement: This study was carried out in strict accordance with the recommendations in the Guide for the Care and Use of Laboratory Animals of the National Institutes of Health. The protocol was approved by the Purdue University Animal Care and Use Committee, protocol #03-095. Euthanasia was performed using CO_2_ inhalation, and all efforts were made to minimize suffering. Intestinal samples (50–100 mg) were taken from chicken embryos at embryonic day (E) 21.5, hatchlings and White Leghorn adult male birds. Tissues were stored in RNAlater, snap frozen in LN_2_ or processed immediately for RNA isolation. Fertile chicken eggs (n = 720) were obtained and incubated (Jamesway Incubator Company Inc., Cambridge, Ontario, Canada) for gene expression studies.

Since intestinal segments can be identified by E14.5, embryonic intestinal tracts (n = 5–8) from E14.5, E15.5, E16.5, E18.5, and E21.5 of incubation and post-hatch chickens (d 1, 3 and 7) were dissected as discussed previously [31]. Intestinal regions include: duodenum (from the ventriculus to the end of the pancreatic loop), jejunum (from the duodenum to the yolk sac), and ileum (from the jejunum to the ileal-cecal junction). Total RNA was isolated using TRIzol® (Invitrogen, Carlsbad, CA). For most studies, 5 µg of total RNA was reverse transcribed with M-MLV (Invitrogen, Carlsbad, CA) using random hexamers. To ensure transcripts of appropriate length, the reverse transcription reaction in studies involving qRT-PCR was performed using the iScript cDNA synthesis kit (Bio-Rad Life Science Research, Hercules, CA), which contains a mixture of random hexamers and oligo d(T). Alternately, some samples were reverse transcribed using oligo d(T) and SuperScript III (Invitrogen, Carlsbad, CA) or SMARTScribe™ (Clontech, Mountain view, CA). Each PCR was performed at least twice to ensure consistency.

### RNA-ligase-mediated rapid amplification of cDNA ends (RLM-RACE)

Total RNA was purified using the DNA-*free™* DNase Treatment and Removal Kit (Ambion Inc., Austin, TX) as described [32]. Integrity was verified by gel electrophoresis (1% agarose, 1× TAE). RLM-RACE was performed using the GeneRacer™ RLM-RACE kit (Invitrogen Inc., Carlsbad, CA) according to the manufacturer's protocol. Briefly, full-length capped mRNA was obtained by treating purified total RNA with calf intestinal phosphatase (CIP), which removes fragmented mRNA and non-mRNA. The protective 5′ cap structure from full-length mRNA was then dephosphorylated with tobacco acid pyrophosphatase to facilitate ligation of an RNA oligo to the 5′ end by T4 RNA ligase. Ligated mRNA (2 µg) was reverse transcribed using SuperScript™ III RT and GeneRacer™ Oligo d(T) primers.

To obtain the 3′ end of the *MUC2* transcript, first strand cDNA was amplified using the provided 3′ anchor primer and a forward, gene specific 3′ primer (GSP). Hot-start *Taq* mixed with *Pfu* polymerase (Advantage© 2 system, Clontech Laboratories, Inc., Mountain View, CA) was used for the 3′ long-range PCR reaction. Amplification was performed under the following conditions: denaturation at 95°C for 1 min, followed by 35 cycles of denaturation at 94°C for 30 s, annealing at 55°C for 1 min, and extension at 68°C for 3 min. To amplify the 5′ end of *MUC2*, a reverse complement 5′-GSP and the 5′ anchor primer from the kit were used for a touchdown PCR with a long DNA polymerase (BIO-X-ACTTM Long Mix, Bioline, Tauton, MA). The conditions for the 5′ touch-down PCR reaction were: 2 min at 94°C for initial denaturation; 5 cycles of 30 s at 94°C followed by 90 s at 72°C; 5 cycles of 30 s at 94°C followed by 90 s at 70°C; 25 cycles of 94°C followed by 30 s at 68°C and 90 s at 70°C; and 7 min at 72°C for the final extension. To obtain the 5′ and 3′ ends, we performed nested PCR on 1 µl of the first round amplification reaction using internal *MUC2*-specific primers for both ends of the transcript and the corresponding anchor primers provided by the kit. RACE products were resolved on 1.2% agarose gels, purified with a gel recovery kit (Zymo Research Corp., Irvine, CA) and cloned using the TOPO TA cloning system (Invitrogen Inc., Carlsbad, CA). Internal primers were designed from either *in silico* sequences or RACE amplified reads. PCR conditions include initial denaturing at 95°C for 5 min followed by 33–34 cycles of denaturation at 94°C for 30 s, annealing at 58 to 63°C for 20 s, and extension at 72°C for 90 to 120 s, and extension at 72°C for 5 min.

### Cloning and sequencing

RT-PCR products were inserted into a pCR-4 TOPO vector and chemically transformed into TOP10 *E. coli* cells (Invitrogen Inc., Carlsbad, CA) as previously described [32]. Long amplicons from RACE-PCR (>2 kb) were cloned into the T vector and chemically transformed into JM109 Competent cells (Promega, Madison, WI). Plasmids from each clone were prepared and purified using a Quicklyse Miniprep kit (Qiagen Inc., Valencia, CA) and digested with *Eco*RI. Digested fragments were resolved by gel electrophoresis on 1.5% agarose, 0.5× TBE gels. Three to ten subclones from each clone were sequenced bidirectionally using BigDye 3.1 on an ABI3730XL apparatus (ABI, Life Technologies). Resulting sequences were aligned using Sequencher™ Software (Gene Codes Corp., Ann Arbor, MI). Additionally, two overlapping EST clones (Accession #s BU287205 and BU368530) downstream to the annotated *MUC2* transcript were purchased (ARK-Genomics, the Roslin Institute, UK) [33] and sequenced as described.

Genomic DNA was isolated from spleen from four independent chicken samples following proteinase K digestion and phenol/chloroform extraction. High molecular weight DNA was collected by spooling and diluted to a concentration of 50 ng/µl for PCR amplification. Following amplification and purification using the DNA Clean & Concentrator™-5 Kit (Zymo Research Corp., Irvine, CA) to remove free nucleotides and excess primers, the amplicons were sequenced using a ¼ BigDye 3.1 reaction. In a 10 µl reaction volume, this corresponds to 2 µl of 5× sequencing buffer, which ensures that the correct concentrations of reagents are included in the sequencing reaction, 5 µM primer, 2 µl of each amplicon, 1 µl of BigDye 3.1 and 5 µl of H_2_O. Sequencing reactions were purified using the ZR DNA Sequencing Clean-up Kit™ (Zymo Research Corp., Irvine, CA) and were sequenced as described above. ABI files were uploaded, aligned and analyzed using Sequencher™ Software (Gene Codes Corp., Ann Arbor, MI).

### Northern blot hybridization

Total RNA prepared from chick intestine was denatured in 50% formamide (v/v), 5% formaldehyde (v/v) and 20 mM MOPS, pH 7.0, at 65°C for 10 min; electrophoresed in 1.2 to 1.3% agarose gels containing 5% formaldehyde (v/v); and transferred to Hybond N^+^ nylon membranes overnight. RNA was fixed by cross-linking under UV for 125 s. Membranes were prehybridized in ULTRAhyb® buffer (Ambion) for 1 h at 42°C. Hybridization was carried out at 42°C overnight in ULTRAhyb® buffer containing ^32^P-labeled probes and 0.1 mg/ml denatured salmon sperm. Probes for chicken *MUC2* were prepared by asymmetric PCR or PCR in the presence of [γ-^32^P]dCTP using gel recovered RT-PCR products as the template. The RNA ladder was radioactively labeled using reverse transcription with random primers. Membranes were washed at 65°C in 2× SSC; 0.1× SDS; 1× SSC; 0.1× SDS, and subsequently 0.1× SSC; 0.1× SDS and exposed to Kodak XAR (Eastman Kodak, Rochester, NY) autoradiography film.

### Quantitative RT-PCR

*MUC2* expression was analyzed by quantitative RT-PCR (qRT-PCR) in embryonic and post-hatch tissues of chicks as described [32]. Primer pairs (Table 1, P34 to P37) for qRT-PCR analysis were optimized, and PCR products were cloned (into the pCR-4TOPO vector) and confirmed by sequencing. Assays were conducted in 15 µL reactions using iQ SYBR Green Supermix (Bio-Rad Life Science Research, Hercules, CA) with diluted first-strand cDNA. qRT-PCR programs for *MUC2* and *18S* RNA were: 5 min at 95°C, 40 cycles of 95°C for 15 sec, 56°C or 57°C for 15 sec, 72°C for 15 sec and 82°C or 83°C for 15 sec data collection, followed by 80 cycles for melting curve analysis. All cDNA samples calculated from 100 ng of total RNA per reaction were assayed in duplicate. Quantification standards were comprised of four 100-fold dilutions of purified plasmid DNA (containing from 10^8^ to 10^2^ molecules or 10^7^ to 10^1^ molecules) and assayed in triplicate with R square values of 0.99 or above. Standards were used to calculate a linear regression model for threshold cycle (Ct) relative to transcript abundance in each sample. The log value of *MUC2* transcript starting abundance was calculated from the Ct values corrected by a factor calculated from *18S* RNA as previously described [31].

**Table 1:** List of Primers.

No	Product ID	Type		Oligo sequence (5′-3′)
P1	*MUC2F1*	RT	F	TTT ATG CTC TGG CTG GCT CTT T
P2		RT	R	GGA GTC CTC ATT TCC TTT ACA TGC
P3	*MUC2F2*	RT	F	ATT GTC ACT CAC GCC TTA ATC TG
P4		RT	R	TTT GTC ATC TAC TAA CAA CAC AAC AGT C
P5	*MUC2F3*	RT	F	ATG TGG TGG TTT TCA GAT CAG ATG
P6		RT	R	AGG TTC CAG ATA TGA CCC CTT GTA
P7	*MUC2F4*	RT	F	TGT GGC TGC CCA GAT AAT ACA TAC
P8		RT	R	CCA TTC CTG CTT GTA AAG TCA TTG
P9	*MUC2F5*	RT	F	TGA TGT GCA TTA CCA GAA CAA GAC
P10		RT	R	ATA TGT CGC CAT CCT TTA TTG TTG
P11	*MUC2F6*	RT	F	TGG TGG AGA AAT ACC AAC AGA AGA
P12		RT	R	TAT TGG TGG TAG GAC TGT GCT TGT
P13	*MUC2F7*	RT	F	CCA CCA CAA GCC AGT CTC CA
P14		RT	R	GCA GTA TGA AAC ATG GCC GTT G
P15	*MUC2F8*	RT	F	TGA AGA ATT AGG GCA GAA GGT TGA
P16		RT	R	GGG CAC TGC TAC TTG ACA CAG TC
P17	*MUC2F9*	RT	F	AAC ACG TAC GAC TGT GTC AAG TAG C
P18		RT	R	GCT GTT GTG CAC TCT GGA CTT AAT
P19	*MUC2F10*	RT	F	ATT AAG TCC AGA GTG CAC AAC AGC
P20		RT	R	GCA CTG CTA CTT GAC ACA GTC GT
P21	*MUC2F11*	RT	F	ACA TTC CTA TAG AAG ATC TAG GGC AGA A
P22		RT	R	CTC CCT CAA CAG GGG AAC AC
P23	*MUC2F12*	RT	F	ACG ACT GTG TCA AGT AGC AGT GC
P24		RT	R	TAC TTG ACA CAC TTG GAC TCG ACA
P25	*MUC2F13*	RT	F	ACC ACC AGT ACA ACA GTG TCG AGT
P26		RT	R	TTC CAC TTT CTG CCC TAG ATC TTC
P27	*MUC2F14*	LR	F	ACA CAG TTC ACC CAC CTT AGC C
P4		LR	R	TTT GTC ATC TAC TAA CAA CAC AAC AGT C
P28	*MUC2F15*	LR	F	AAC GGC AAC TGA AAT AGT CTG CAC CTT C
P29		LR	R	AAT GTG CTT TTA ATC ATT CAG AGA AAA TAA GTT GAT T
P30	*MUC2F16*	LR	F	CGG GCC AAC ACC TAC CAC CTC
P29		LR	R	AAT GTG CTT TTA ATC ATT CAG AGA AAA TAA GTT GAT T
P31	*MUC2F17*	LR	F	TAA CTC AAA CCC ACT CTC CTC CAC CTT C
P29		LR	R	AAT GTG CTT TTA ATC ATT CAG AGA AAA TAA GTT GAT T
P19	*MUC2F18*	LR	F	ATT AAG TCC AGA GTG CAC AAC AGC
P22		LR	R	CTC CCT CAA CAG GGG AAC AC
P32	*MUC2F19*	5′RACE	R	CTA ACA CAT GGA AAG CTC AGC CCA CC
P33	*MUC2F20*	3′RACE	F	CGG GCC AAC ACC TAC CAC CTC
P34	*MUC2RQ*	qRT	F	ATT GTG GTA ACA CCA ACA TTC ATC
P35		qRT	R	CTT TAT AAT GTC AGC ACC AAC TTC TC
P36	*18S*	qRT	F	GCC ACC CGA GAT TGA GCA ATA ACA
P37		qRT	R	TAG ACA CAA GCT GAG CCA GTC AGT
P38	*HPRT*	RT-PCR	F	ATG ACC ACT GTC CAT GCC ATC
P39		RT-PCR	R	AGG GAT GAC TTT CCC TAC AGC CTT
P40	ARK clone	Seq	F	GGA GAG AGT TGT CCT GAC TGA ATG
P41	ARK clone	Seq	R	CAC AAG AGA AGA GCC ATC AG
P42	*MUC2F15,16,17*	Seq	R	TCC AGG TCT AAG TCG GGA AGT G
P43	*MUC2F15,16,17*	Seq	F	CAC CTC CTA AAC CCA CCT GCT
P44	*MUC2F15,16,17*	Seq	R	CCG CAG CTT TCC ACA TAC AC
P45	*MUC2F15,16,17*	Seq	F	GTG TTT GAG AAG TGC CGT GAA G
P46	*MUC2F15,17*	Seq	F	ACA CTC AAC CAC TAC AAC CAT
P47	*MUC2F15,17*	Seq	R	AAG GTA ATT GTC TGG CCG TGG TG
P48	*MUC2F15*	Seq	F	CCT GTT AAC ACA CAG TCT ACA GGA G
P49	*MUC2F15*	Seq	F	GCT CTT CAA CAG CTT CAG TTT
P51	*MUC2F15*	Seq	R	GGC TCA CAG ATT ACT GGA ACG A
P52	*MUC2F15*	Seq	R	ATT GGA GCA GGT GGG TTT AGG
P53	*E45F*	DNA	F	AGA GCT CTC AGA CAC AGT GGT TGT
P54	*E46R*	DNA	R	CAT TTT CCA TGA GCT CCC TTA CTT

RT-PCR (RT); long-range PCR (LR); quantitative RT-PCR (qRT); sequencing (Seq); Forward (F); Reverse (R).

## Results

### Cloning the chicken MUC2 cDNA

**Figure 1:**
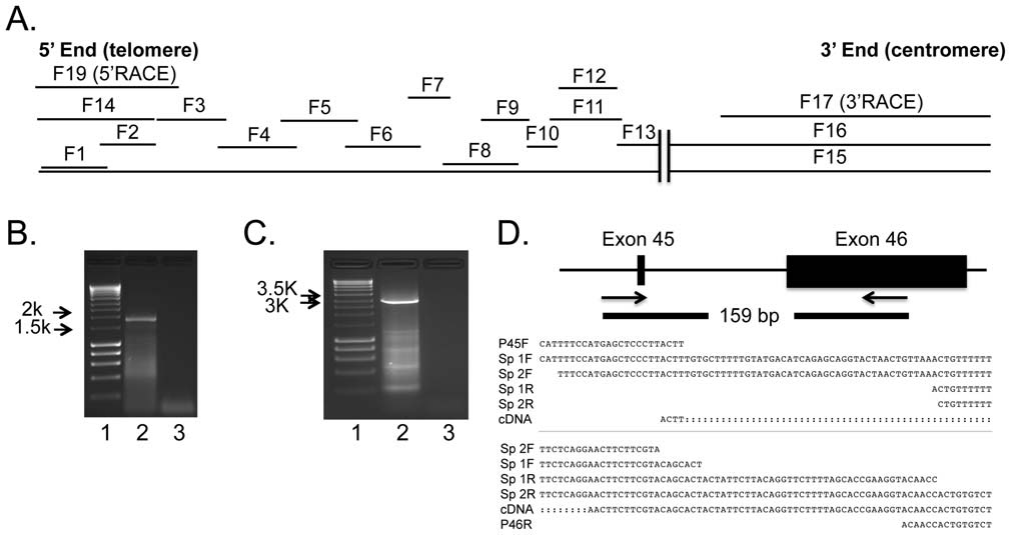
*MUC2* cDNA contig. A. *MUC2* cDNA contigs. Sixteen overlapping RT-PCR amplicons and two RACE products are depicted. The double vertical lines represent the cDNA gap in the PTS domain. EST sequences are not shown. B. 5′ RACE. Representative image from 5′ RACE products size fractionated on a 1.2% 1× TAE gel. Molecular weight marker (Lane 1); 5′ RACE produce (Lane 2); Water control (Lane 3). C. 3′ RACE. Representative image from 3′ RACE products size fractionated on a 1.5% 1× TAE gel. Molecular weight marker (Lane 1); 3′ RACE produce (Lane 2); Water control (Lane 3). D. Genomic PCR. We amplified a 159 bp fragment that spanned the gap between exons 45 and 46. Exons are depicted to scale. The forward and reverse sequences from two spleen (Sp 1 and Sp2) samples are shown, along with the cDNA sequence for exons 45 and 46 and the primers. The last 9 bp from the reverse primer are not shown.

In our aim to clone the full-length chicken *MUC2* gene, we amplified, cloned and sequenced 16 overlapping *MUC2* RT-PCR products (F1–F14), two expressed sequence tags (ESTs) from the 3′ end of the *MUC2* gene (not shown), and products from 5′ and 3′ RACE (F17, F19) (Figure 1A). We sequenced the 1.5 kb 3′-RACE and 3.3 kb 5′-RACE clones in their entirety using multiple internal primers (Figure 1B and 1C). RT-PCR clones derived from internal primers were sequenced to confirm the exon-intron junctions of the 5′ RACE product (F14, Figure 1A). Long-range RT-PCR was performed to determine the sequence of the central and 3′ terminal exons of *MUC2*, resulting in amplification of two fragments close to 3.7 kb in size (F15 and F16, Figure 1A). We sequenced two overlapping EST clones (Accession #s BU287205 and BU368530) [33] located at the 3′ end of the cDNA in an attempt to close the gap (Figure 1A) produced by the highly polymorphic PTS domain, however this was not successful..

To close the gap, we used BLAT alignment [34] to map the cDNA to the UCSC reference sequence (Nov. 2011 (ICGSC Gallus_gallus–4.0/galGal4)) [28]. Based on the genomic alignment, the UCSC database predicted that there was no gap in the cDNA, as the two exons spanning the gap (Table 2, exons 45 and 46) were located within a 136 bp sequence with a short intron. To confirm the genomic organization, we designed primers that flanked the putative gap in the genome. PCR amplification and sequence analysis confirmed that the UCSC annotation was correct, and that we had spanned the gap between the two cDNA contigs (Figure 1D).

**Table 2:** Genomic Structure of Chicken *MUC2*.

Exon	cDNA		Exon	UCSC Alignment^1^		Intron	Splice Junctions	
#	Start	Stop	length	Start	Stop	length	5′ Splice acceptor	3′ Splice donor
1^a^	1	94	94	14368918	14368825	1490	cagtggtattCACAGTTCAC	ATAAGAAAAGgtaagctcta
2	95	359	265	14367335	14367071	2588	aacttaacagGAAGGACAAG	ATGGGCGGATgtaagtacat
3	360	474	115	14364483	14364369	698	ttcttcctagTGTCAAGACA	TGCACTGATGgtatgtaaaa
4	475	565	91	14363671	14363581	411	cattacctagGTGGAGCTGG	ATTAGTGGAGgtgagtcata
5	566	675	110	14363170	14363061	237	tttctcacagTTGCAAGCTA	TAATGAGCATgtgagtaagc
5^b^	566	744	179	14363060	14362992	168	taatgagcatGTGAGTAAGC	TGAGATGTTAgtgtgtgaag
6	745	961	217	14362824	14362608	450	tccttcccagCGTGAGGAAT	AACTTTTGCTgtaagcagct
7	962	1087	126	14362158	14362033	260	tttttttcagACAAAACATG	TGCCCCGAAGgcaagtgtat
8	1088	1205	118	14361773	14361656	318	tgctttacagGGACTGTGTA	GTGAAGAATGgtaaggatca
9	1206	1344	139	14361338	14361200	663	ttgtttatagCACCTGTGAT	GTTGGCCAAGgtactgtata
10	1345	1455	111	14360537	14360427	1040	gtgattgtagAGTGCTGTGA	CAAAAAAAATgtaagtgttg
11	1456	1525	70	14359387	14359318	1334	ttttatacagGTGGTGGTTT	CATGTGTCAGgtaaggacca
12	1526	1660	135	14357984	14357850	527	tgtttttcagCTAGCTTCTC	GACATTCAAGgcaagtgtcc
12^b^	1526	1735	210	14357849	14357774	451	acattcaaggCAAGTGTCCA	ACATGGGTGGtaattcatag
13	1736	1900	165	14357323	14357159	608	tttttgatagGTCTTTGTGG	ATTGAAAGTGgtaagcttgc
14	1901	1995	95	14356551	14356457	623	ttttttgcagCAAATTATGC	ATACTATAAGgtatggtaat
15	1996	2122	127	14355834	14355708	578	gctttttcagAGATGTAAAT	AGTGTCTGCTgtaagtaatg
16	2123	2378	256	14355130	14354875	854	tcccctccagCTGATGAAGT	GAGAACGTTGgtatgtgtta
17	2379	2431	53	14354021	14353969	289	tctttcctagTGTTTGCCGA	AGAATGAAAAgtaagtgaca
18	2432	2541	110	14353680	14353571	435	taactttcagGTATAACAGA	AACTGATCTTgtatgtattc
19	2542	2693	152	14353136	14352985	433	tgctccttagTATCAAGGTG	GCAACACCTGgtatgctggt
20	2694	2832	139	14352552	14352414	543	tctcttctagTACCTGCCAG	GGCTACTCAGgtaatgctca
21	2833	2943	111	14351871	14351761	342	ttatccacagGATTATTGCG	GTTTATAGGGgtgagtaatg
22	2944	3123	180	14351419	14351240	995	taccatttagAAAACTGAAC	TGACTATAAAgtaagttaga
23	3124	3363	240	14350245	14350006	1149	ccttttgcagGGGAAAGTGT	TCATTCCAAGgtttgtagat
24	3364	3520	157	14348857	14348701	1075	tttccaacagGTGAACCCAT	GATATATGCCgtgagtaaca
25	3521	3649	129	14347626	14347498	1713	ttctcatcagCAATATTCTG	TACCTGGAAGgtaataaatt
26	3650	3797	148	14345785	14345638	911	gcatcaatagGTTGCTACCC	GTACAAAATGgtatgtaaaa
27	3798	3841	44	14344727	14344684	473	ttgcttttagTATCTGTCGC	CCAATTCCAGgtaaatagtg
28	3842	4039	198	14344211	14344014	354	tttcttgcagGATGTCCTTG	ACCACCATAGgtaagttttc
29	4040	4066	27	14343660	14343634	350	tctatttcagTTACCACAAG	GTACCTACAAgtaagttttg
30	4067	4543	477	14343284	14342808	852	tttcttgcagCTCCATGCCT	GGCTCCACAGgtatttagca
31	4544	4735	192	14341956	14341765	1072	tctcttccagTATCAACCAC	TCGCAACCAGgtgattaatt
32	4736	5338	603	14340693	14340091	1025	tgtctctcagTAGGAAATTG	TCGACAGAAGgtaattgtct
33	5339	5577	239	14339066	14338828		ttttccacagGTCCCACTCC	GACAACTGAAggtaatgtct
34^c^	5578	5775	198				nnnnnnnnnnATAGTCTGCA	AGAAGATCTAnnnnnnnnnn
35	5776	5786	11	14338698	14338688		tgtttgtcttGGGCA	GAAAGTcccttagtc
36^d^	5787	6209	423				nnnnnnnnnnGGAATGCGAT	CGTCGACAGAnnnnnnnnnn
37	6210	6453	244	14336621	14336378		gtgttatcccAGGTCCCACT	GACAGAAGGTaattgtctgg
38^c^	6454	6497	44				nnnnnnnnnnCCCACTTCCC	CGAGTCCAAGnnnnnnnnnn
39^e^	6498	6675	178	14335163	14334986		cacgtacgacTGTGTCAAGT	ACCCACCACCnnnnnnnnnn
40^d^	6676	6704	29				nnnnnnnnnnTCCGTAACAC	CGTCGACAGAnnnnnnnnnn
41	6705	6946	242	14313408	14313167	1173	gtgttatcccAGGTCCCACT	TCGACAGAAGgtaattgtct
42	6947	7203	257	14311994	14311738		gttttcccagGTCCCACTTC	GACAGAAGGTaattgtctgg
43^c^	7204	8072	869				nnnnnnnnnnCCCACTCCTG	CGTCGACAGAnnnnnnnnnn
44	8073	8359	287	14310552	14310266	1463	gtgttatcccAGGTCCCACT	TTGAGGGAGAggtgaaggcc
45	8360	8363	4	14308803	14308800		gagctcccttAC	TTtgtgcttttt
46	8364	8823	460	14308740	14308281	1002	ttttctcaggAACTTCTTCG	AACTCATGCGgttagtgaat
47	8824	8998	175	14307279	14307105	566	ccaaatacagCCGGGTGAGT	GAGTGTGATTgtaagtatat
48	8999	9246	248	14306539	14306292	888	gtttttgcagGCTACTGCAC	GGAAGTAGAGgtattggaga
49	9247	9430	184	14305404	14305221	134	tatttactagGTGACTGTAA	GGACAGTGTGgtaaggctta
50	9431	9635	205	14305087	14304883	644	cccattttagGCATTTGCAA	TTTGGGGAAGgtaggcatgc
51	9636	9814	179	14304239	14304061	176	ctgtttttagTGTGTTTGAG	GGTGTTTGCTgtaagtattt
52^f^	9815	9817	3	14303885	14303883	1879	ccgctctctgGC	Tgtgtgtgtgg
53	9818	9887	70	14302004	14301935	315	cccattccagCTTATGAATG	GCAAGTCAAGgtaaagaatt
54^b^^,^ f	9888	9988	101	14301620	14301520	157	tctcatacagTCCACAAAA	AATACCTGTGgtgagtttta
55	9989	10020	32	14301363	14301332	157	attttcacagGCTGTGTTGG	ACCAAGAGAGgtacgctgcc
56	10021	10198	178	14301175	14300998	707	ttaatgacagTTTGGAGAAA	GTCACTTGCAgtgagttaat
57	10199	10303	105	14300291	14300187	576	tttttttcagAATGCAACAC	TACAAGTGTGgtaagtcctt
58	10304	10344	41	14299611	14299571	644	ttgttaacagTTCCCAAGAA	TGAGTTCTTGgtaagttaat
59	10345	10467	123	14298927	14298805	452	aaatttatagCCTAATTCCT	TTGTGAACCAgtaagtgatg
60	10468	10566	99	14298353	14298255	625	taatatctagGGATATGAAC	CATACTGAATgtaagtatgc
61	10567	10695	129	14297630	14297502	665	tcacttgcagCCTGGAGAGT	CTGCAAACCTgtaagtagat
62	10696	10735	40	14296837	14296798	289	gttcttttagGGAACTGTTA	TGCAAAACCTgtaagtatgt
63	10736	10865	130	14296509	14296380	1513	ttccctttagGTATACCTCT	CTTTCTCACTgtaagtaaac
64^g^	10866	11359	494	14294867	14294374		atcttaacagGTACTCTGTT	ATTAAAAAAAaaaacatggg

1 Nov 2011 Build (ICGSC Gallus_gallus-4.0/galGal4).

a Translational start site at nucleotide 25.

b Predicted alternate splicing events (exons 5b & 12b) or exons (exon 55) from reference [3] that were not cloned in this study.

c Assembly error—Exon is missing from the current assembly.

d Assembly error—Exon located in Gap.

e Assembly error—Exon ends in Gap.

f Sequence was found in cerebrum and in primordial germ cells in the embyronic gondal, but not cloned in intestine in this study.

g Translational stop site at nucleotide 11117.

We next assembled all of the cDNA clones, as well as the predicted cDNA and annotated mRNAs and ESTs from the UCSC and NCBI databases into an 11,359 bp chicken *MUC2* cDNA sequence (Figure S1), which has been deposited into GenBank (Accession # JX284122). Translation of the cDNA indicates that we identified a 3697 amino acid protein (Figure S2), which is 1482 amino acids shorter than the predicted human orthologue [5] and 1017 amino acids longer than the annotated mouse protein [24], [35].

### MUC2 genomic organization and protein structure

**Figure 2:**
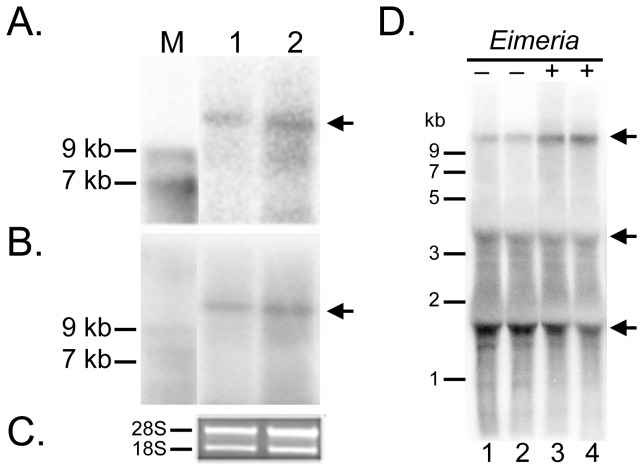
Northern blot analysis of intestinal *MUC2*. A. Transcripts detected with a 5′ probe. B. Transcripts detected with a 3′ probe. Arrows show that both probes detected the same band. Membranes were stripped and demonstrated to show no signal prior to rehybridization. Lane M, ssRNA ladder (kilobase); Lane 1 and 2, 20 µg total RNA per lane. C. RNA integrity. Ethidium bromide-stained rRNAs of the same RNA samples electrophoresed on a 1.0% TAE gel are shown as an internal control. D. *MUC2* expression following *Eimeria* infection. *MUC2* transcripts detected in total RNA (30 µg) from cecal tonsils of chickens infected with *Eimeria* protozoa (+, lanes 3 and 4) or not (−, lane 1 and 2) were identified by probe 3 (1329-bp), which was synthesized by asymmetric PCR. Each lane represents pooled samples from 4 to 5 chickens. Arrow head: *MUC2* mRNA.

**Figure 3:**
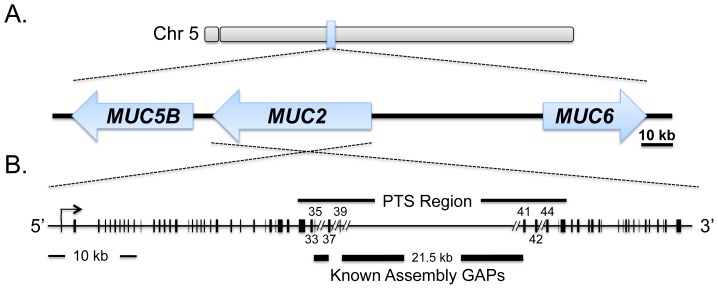
Chicken *MUC2* physical map and genomic organization. A. Physical Map. The *MUC2* locus lies on chromosome 5 between *MUC5B* and *MUC6*, and spans 74.5 kb of genomic DNA. B. Gene Structure. We identified an 11,359 bp *MUC2* cDNA. Black vertical lines represent the location and relative size of the exons. There are two known assembly gaps in the chicken genome, based on the UCSC Nov 2011 Build (ICGSC Gallus_gallus-4.0/galGal4). Exon 36 (423 bp) is located in the smaller gap, and therefore not included in this figure, as it is unknown where it lies in relationship to its flanking exons. Exon 39 ends blindly in the large (∼21 kb) assembly gap. Exon 40 (29 bp, also not drawn in this map) is located within the gap, and exon 41 is intact on the other side of the gap. The location of these gaps are denoted by large black bars under the gene structure, and gaps are denoted in the genomic structure, when possible (the space between exons 33 and 35 is too small to insert a gap). Exons 34 (198 bp), 38 (44 bp) and 43 (869 bp), which are also not included in this figure) do not fall within the annotated gaps, but are missing in the genomic sequence, indicating errors in the assembly.

Using northern blot analysis, we estimated the size of the full-length *MUC2* transcript to be approximately 12 kb using probes targeting the 3′ and 5′ termini (Figure 2A and 2B). *MUC2* is expressed from the (–) strand and spans 74.5 kb of genomic DNA (nucleotides 14368918 to 14294373) on chicken chromosome 5 (Table 2; Figure 3A, 3B). Alignment of our *MUC2* cDNA with the Nov 2011 Build (ICGSC Gallus_gallus–4.0/galGal4) of the chicken reference genome [28] indicates that *MUC2* spans at least 64 exons (Table 2; Figure 3B). The translational start site occurs within exon 1 at nucleotide 25, while the translational stop site is found at position 11,117 in exon 64.

**Figure 4:**
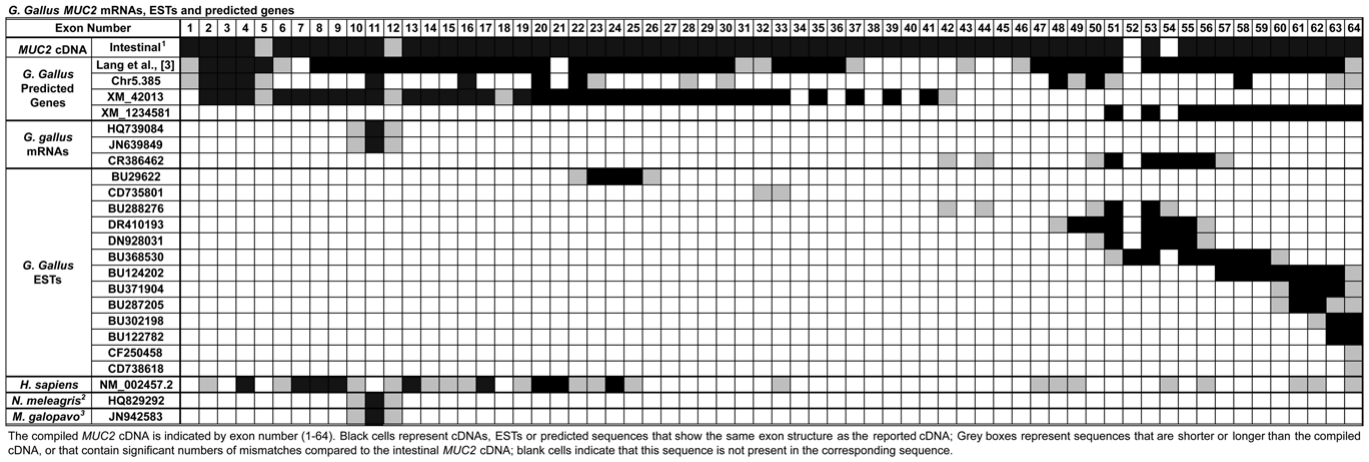
Functional Annotation of *MUC2*. The compiled *MUC2* cDNA is indicated by exon number (1–64). Black cells represent cDNAs, ESTs or predicted sequences that show the same exon structure as the reported cDNA; Grey boxes represent sequences that are shorter or longer than the compiled cDNA, or that contain significant numbers of mismatches compared to the intestinal *MUC2* cDNA; blank cells indicate that this sequence is not present in the corresponding sequence. ^1^ *G. gallus* intestinal *MUC2* cDNA from this report; ^2^Helmeted guineafowl; ^3^Turkey.

By comparing the positions of known chicken mRNAs, ESTs and predicted transcripts, as well as cross-species comparison of human, turkey and helmeted guineafowl mRNAs with our cloned cDNA, we demonstrate strong evidence for our annotation of the genomic structure of *MUC2* in chicken (Table 2, Figure 4). Three partial chicken *MUC2* mRNAs share significant overlap with our gene. HQ739084 (derived from spleen) and JN639849 share perfect homology with exons 9–11, while CR386462 overlaps with exons 42, 44, 50, 51, 53, 54–57. However, exons 54–56 are annotated as one exon in cDNA CR386462, and exon 57 is smaller than the sequence we cloned. Several chicken ESTs map to our *MUC2* exons and add two additional exons. BU296220 overlaps with exons 23–27, while CD753801 maps to exons 32 and 33. BU288276 aligns with exons 42, 44, 50, 51, 53, 54 and DR410193 shares sequence identity with exons 48–51, 53–56. DN928031 maps to exons 50, 51, 53–56; BU368530 overlaps with exons 51–53, 55–60; and BU124202 has significant overlap with exons 57–64. BU371904 and BU287205 share exons 60–64; BU302198 lies within exons 62–64; BU122782 is located within exons 63 and 64; and CF250458 and CD738616 map to exon 64. The human *MUC2* transcript overlaps with exons 2, 4, 6–10, 12–17, 19–25, 33, 47–49, 54, 56, 61, 62 and 64, while the helmeted guineafowl (HQ829292) and the turkey (JN942583) transcripts share homology with exons 9–11.

Four predicted transcripts provide additional support for our *MUC2* gene structure (Figure 4). The most complete predicted chicken sequence [3] overlaps with exons 1–6, 8–20, 22–37, 43, 46–51, 53–64. However, exons 1, 6, 31, 32, 37, 43, 46 and 64 are shorter than the cloned intestinal sequences. The GeneScan predicted gene, *Chr5.385*, overlaps exons 1–5, 11, 16, 21, 23, 24, 28, 30, 48–51, 58, 63 and 64 of our cDNA. Significant overlap occurs with two additional predicted *MUC2* chicken cDNAs (XM_421035 and XM_001234581) that have been annotated in GenBank (www.ncbi.nlm.nih.gov/nucleotide/) [36]. Although XM_421035 has been removed, BLAT analysis of the sequence aligns perfectly with exons 2–31 of our cloned *MUC2* cDNA, while XM_001234581 aligns directly with exons 51, 53, and 55–64 of our annotated *MUC2* cDNA.

The alignment of our cDNA, along with the chicken mRNAs, ESTs and putative transcripts, to the annotated genome matches very well between exons 1–33 and 46–64. However, there are several inconsistencies between exons 34 and 46 (Table 2; Figure 3B). Exons 34, 38 and 43 are completely missing from the assembly, while exon 36 is located within the small gap and exon 39 ends abruptly in the large 21.5 kb gap (Figure 3B). In an attempt to close these gaps, we designed primers that spanned exons 33–35. The predicted amplicon from this region is 2,340 bp. Despite repeated efforts, we were unsuccessful in generating the correct amplicon, due to the fact that the genomic DNA flanking both of these exons contains several elements that are repeated between exons 32 through 39 and exons 41 through 44. In addition, the presence of multiple poly T and poly A tracts within these regions hampered amplification and/or sequencing efforts due to slippage of the polymerase. Similar challenges occurred when we tried to design primers to amplify the region between exons 37 and 39 and between exons 42 and 44.

The chicken *MUC2* locus contains a 21,496 bp gap in the assembly. When we align the compiled cDNA to the genomic locus, we were surprised to discover that the only exon that falls within this large gap is exon 40 (29 bp). Since exon 40 lies within the highly repetitive PTS domain, attempts at cloning the intervening sequences by PCR of genomic DNA have been unsuccessful. Similar challenges occur in the human and mouse genes, and it is likely that additional exons in this region could be identified when the technology becomes available to sequence long DNA or cDNA molecules, as assembling DNA or cDNA that contains multiple repeated cassettes is a major challenge with the current Sanger sequencing and next generation sequencing technologies.

### Expression analyses of MUC2

**Figure 5:**
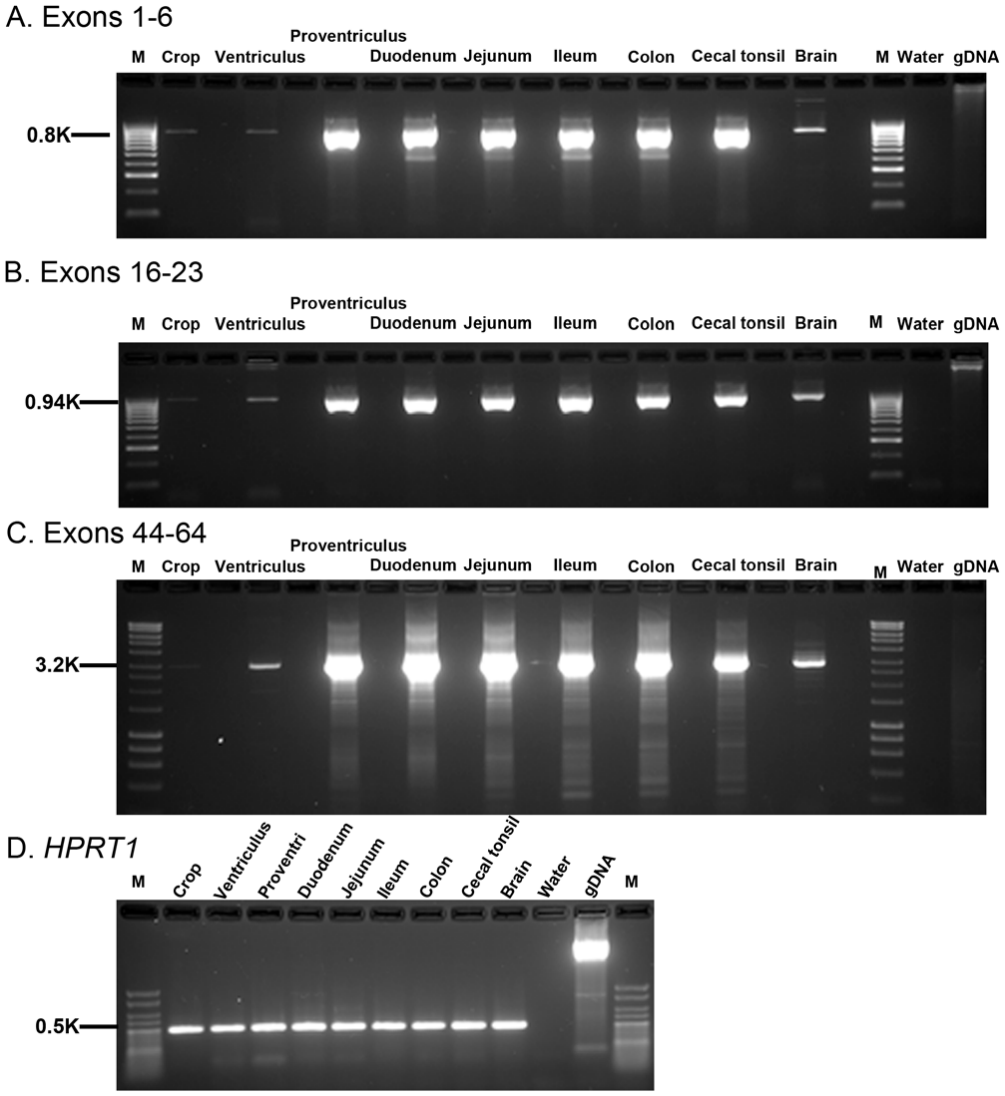
Expression of *MUC2* in the gastrointestinal tract and brain. RNAs were reverse transcribed using SMARTScribe™ (Clontech) with Oligo d(T) to generate long, full-length cDNA. We performed 33 cycles of RT-PCR amplification on 40 ng of cDNA with three sets of *MUC2* primers. Alternating blank lanes lack reverse transcriptase. A. Exons 1–6. *MUC2* is highly expressed in the proventriculus, duodenum, jejunum, ileum, colon, and cecal tonsil, with lower levels in the brain and minimal expression in the crop and ventriculus. Although these primers (P27 and P2) amplify genomic DNA, Genomic DNA controls demonstrate the lack of genomic contamination in all samples, indicating that observed expression is from cDNA B. Exons 16–23. *MUC2* is highly expressed in the proventriculus, duodenum, jejunum, ileum, colon, and cecal tonsil, with lower levels in the brain and minimal expression in the crop and ventriculus. Although these primers (P7 and P8) amplify genomic DNA, Genomic DNA controls demonstrate the lack of genomic contamination in all samples, indicating that observed expression is from cDNA C Exons 44–65. A touchdown long-range PCR was used to amply the 3′ end of *MUC2* using an internal primer and a primer targeting the exact end of the *MUC2* cDNA (P30 and P29). This region demonstrates a very similar pattern of expression, with high levels detected in all tissues, except brain, which shows low-level expression,and ventriculus which has minimal expression. No expression is detected in the crop in this analysis. D. *HPRT1* control gene. All samples express *HPRT*, and lack the presence of the genomic DNA band, indicating that the samples do not have genomic contamination. RT-PCR products were examined by electrophoresis through a 2.5% agarose gel in 0.5× TBE (A and B) or 1.2% TAE; water and genomic DNA were used as controls.

**Figure 6:**
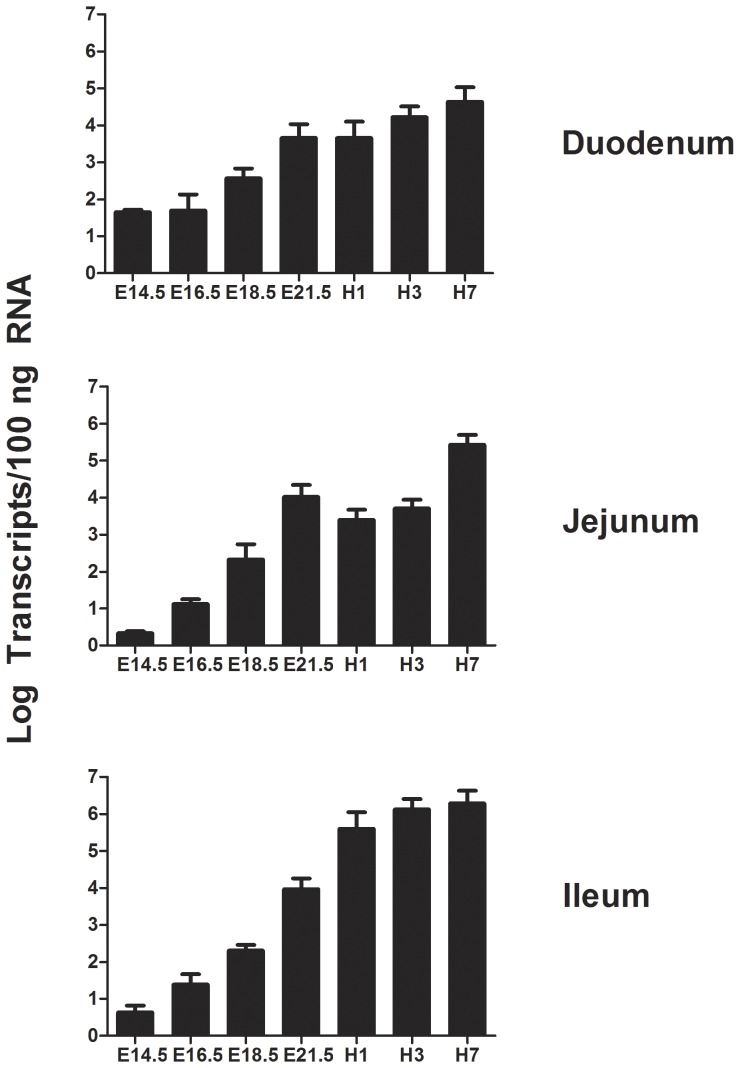
Temporal expression of *MUC2* transcripts. Quantification of *MUC2* transcripts in embryonic (E) and post-hatch (H) ages in duodenum (top), jejunum (middle) and ileum (bottom) using qRT-PCR. *MUC2* expression was normalized to *18S* RNA. The *MUC2* primers hybridize to exons 25 (P35) and 26 (P34) and generate a 135 bp product that spans intron 25 (1710 bp).

We investigated spatial expression of *MUC2* throughout the gastrointestinal tract by RT-PCR and temporal expression in the small intestine at embryonic (E) days 14.5, 16.5, 18.5, 21.5 of incubation and 1, 3, 5 days post-hatch by qRT-PCR (Figures 5 and 6). We used amplicons that spanned three distinct regions of the gene (Exons 1–6; 16–23 and 44–64). *MUC2* is highly expressed throughout the gastrointestinal tract, with weak signals in the crop and brain (Figure 5A, 5B and 5C). We observed no alternative splicing using any of these primer pairs. Quantitative RT-PCR analysis of intestinal *MUC2* (Exons 25–26; primers P34 and P35) during embryogenesis indicates that expression initiates during late embryogenesis, increasing as development progresses (Figure 6). In the duodenum, jejunum and ileum, *MUC2* mRNA levels are relatively low at E14.5, and steadily increase through E21.5. Expression of *MUC2* at E14.5 was further confirmed by gel electrophoresis (data not shown). At day of hatch (E21.5), relative *MUC2* mRNA levels show a spike (1 to 2 logs) in duodenal and ileal tissues, followed by a steady increase throughout the post-hatch time points. In the jejunum, *MUC2* mRNA levels surge to an approximate 2-log increase at H1 followed by a decrease from 1 to 3 d post-hatch, and remain high at 7 d post-hatch.

### Alternative splicing of chicken MUC2

**Figure 7:**
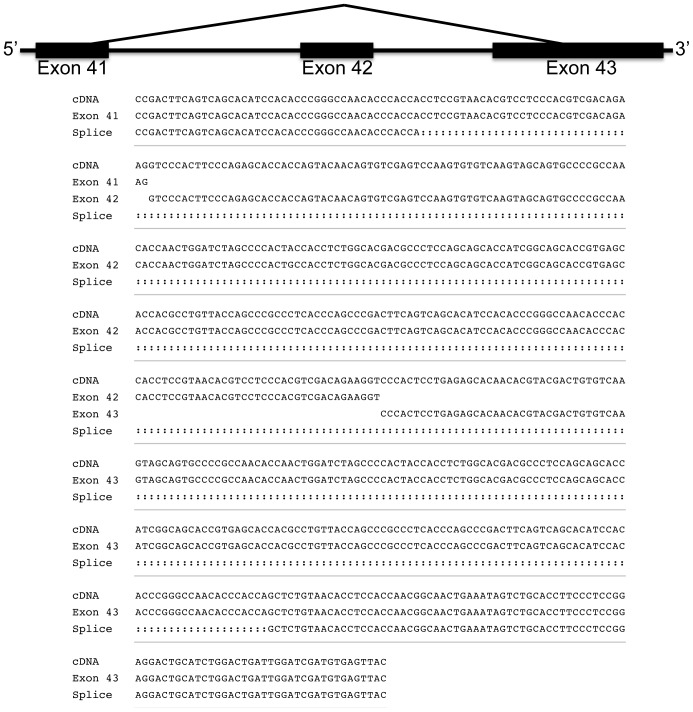
Alternate splicing. We characterized one of the alternatively spliced products and determined that it was generated from an internal 3′ splice donor in exon 41. This transcript skipped exon 42, and used an alternate splice acceptor site in exon 43.

We investigated the presence of alternative splicing events of *MUC2* by RT-PCR, long-range PCR and available ESTs. Several sets of primers spanning the entire cDNA were assayed in multiple tissue types. We identified and characterized one distinct splicing event (Figure 7); we detected one shorter fragment in cecal tonsil samples, which revealed that this transcript used internal splice acceptor/donor sites in exons 41 and 43, removing exon 42. This product is 495 bp shorter than the full-length transcript, but is predicted to result in an in-frame deletion of 165 amino acids within the central PTS domain. Moreover, to explore whether massive alternative splicing events of *MUC2* gene would occur in infected versus normal intestine, *MUC2* transcripts in *Eimeria* infected chicks were analyzed, as *MUC2* has reported to be aberrantly expressed and critically involved in the pathogenesis of coccidiosis [20], a prevalent protozoal disease in the gastro-intestinal tract of the chicken. However, no detectable alternative splicing event(s) were observed at this the resolution (Figure 2D).

### Predicted amino acid sequences and protein structure

**Figure 8:**
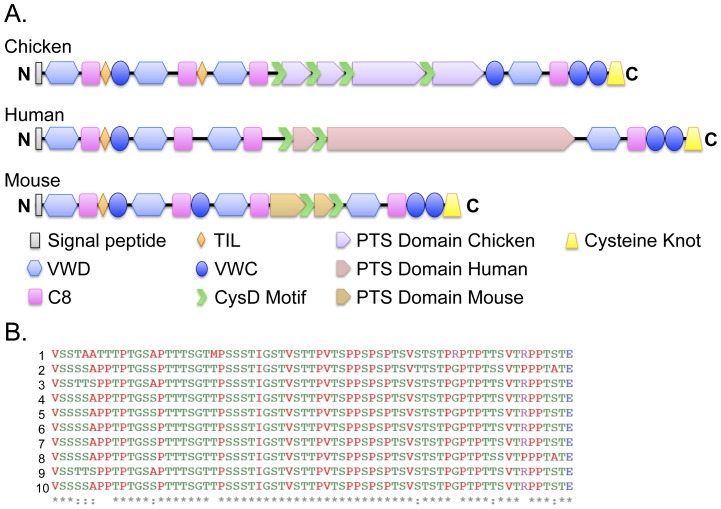
Cross-species comparison of the MUC2 protein structure. A. Protein structure of chicken, human and mouse MUC2. InterProScan protein domain prediction analysis (www.ebi.ac.uk/Tools/pfa/iprscan/) [37] indicates that the full-length chicken intestinal *MUC2* transcript encodes a 3697 amino acid (aa) protein with a short signal peptide at the N-terminus, multiple von Willebrand factor domain structures (VWD, VWC), several cysteine-rich domains (C8), two trypsin Inhibitor-like cysteine-rich domains (TIL), a 1614 amino acid central PTS domain that is interspersed with four CysD motifs and a C-terminal cystine knot (CT). The structure of the human (5179 aa) and mouse (2680 aa) proteins shows strong homology on both sides of the central PTS domain. The two exceptions are that humans and mice lack the second TIL domain, and mice have an additional VWC motif. Although the N-terminal and C-terminal sequences are highly conserved amongst species, the PTS domain is highly divergent, containing different types and varying numbers of repeat cassettes within the central domain. In chicken, this region stretches between aa 1308 and 2922. The different colors in the cartoons represent the finding that the PTS domains are highly divergent among the three species. B. Sequence comparison of the 10 repeats within the PTS domain. Amino acids 1702 through 2763 demarcate this highly repetitive element, which spans exons 32 through 45. RADAR analysis (http://www.ebi.ac.uk/Tools/Radar/) [38] indicates that these cassettes consist of three blocks of repetitive elements interspersed with two CysD domains. Each repeat is 69 amino acids in length and contains one of two short spacer motifs. Repeats 1 and 2 are located in block one, repeats 3–8 are located in block two and repeats 9 and 10 are located in block 3.

We used a combination of protein analysis software (Interproscan; http://www.ebi.ac.uk/Tools/pfa/iprscan/) [37] and analysis of the domain structure of the predicted protein by The Mucin Biology Group (http://www.medkem.gu.se/mucinbiology/databases/) to determine the putative domain structure of chicken MUC2 (Figure 8A). The deduced amino acid sequence of MUC2 contains several elements common to gel-forming mucins, including: VWD and VWC regions; a central PTS domain interspersed with CysD motifs; and a characteristic cystine knot (CT) at the C terminus (Figure S2). Other prominent features include four cysteine-rich regions (C8) and two trypsin inhibitor-like, cysteine rich domains (TIL).

We used Rapid Automatic Detection and Alignment of Repeats (RADAR) profiling (http://www.ebi.ac.uk/Tools/Radar/) [38] to detect a core repetitive cassette within the PTS domain. There are 10 total cassettes within this region in chicken, which encompasses amino acids 1702 through 2763 (nt 5131 through 8313) and spans exons 32 through 44. These 10 cassettes are split into three regions containing varying numbers of a highly similar 69 amino acid repetitive element (Figure 8B): element one contains two repeats interspersed with a GPTPESTTRTT motif; element two contains 6 repeats interspersed with alternating GPTPESTTRTT and GPTSQSTTSTTVSSPS motifs; while element three contains two repetitive cassettes with a GPTPESTTRTT linker motif. These three regions are divided by two of the four CysD domains.

Although the N-terminus and the C-terminus share significant identity among human, mouse and chicken, the PTS domain is highly divergent amongst these three species. The human MUC2 protein contains two types of PTS motifs. The larger one contains 97 highly identical direct head to tail repeats of a 23 amino acid sequence (PTGTQTPTTTPITTTTTVTPTPT). The PTS domain in the mouse is separated in two clusters; cluster one contains nine imperfect duplications of an 8 amino acid repeat, while section two contains 15 imperfect duplications of a 10 amino acid cassette [24].

## Discussion

It has been over two decades since the initial cloning of the first intestinal mucin gene in humans [39]. Although the physiological implications and disease associations of mucins on various mucosal surfaces have been well recognized, many questions remain as to how and why the gene architecture of this family contributes to diverse protein modifications that may display distinct functionalities. Different species demonstrate structural and sequence conservations as well as their own uniqueness. Chicken, the most-studied and characterized avian species, bridges the evolutionary gap between mammals and non-amniote vertebrates, providing an excellent model system for agricultural and biological research.

In the mucin family, the PTS-domains (or mucin domains) are highly polymorphic in both length and sequence in humans, which is primarily due to the presence of multiple alleles of various number of tandem repeats (VNTRs). However, the presence of the VNTR, as well as the cDNA sequence within the PTS domain, is not highly conserved evolutionarily [3], highlighting the distinct possibility that broad functional differences exist between species [40]. Our data indicate that the PTS domain of the chicken MUC2 protein contains a vastly different repeat structure than the human protein. Although the chicken PTS region is shorter, the central repeat motif is 69 amino acids in length (as opposed to a 23 amino acid cassette in humans) and shows very little identity with the human motif.

Recent *in vitro* studies using human intestinal cells demonstrated that the intestinal mucins isolated from chicken were detrimental to the proliferation of *Campylobacter jejuni*, an infectious bacteria causing acute gastroenteritis in humans but not in chicken [10], [40], [41]. In addition these studies demonstrated that the chicken mucins attenuated the invasiveness of *Campylobacter jejuni*, suggesting that differences in mucin protein sequence or structure between humans and chicken could account for the differences in susceptibility to infection. Alternatively, the functional differences between human and chicken may imply species-specific divergence in intestinal mucus composition and/or structures. This could also occur through differences in posttranslational modifications of the human and chicken proteins. Outside of the PTS domain, the human and chicken MUC2 proteins share large blocks of highly conserved sequences, strongly suggesting that this variable PTS region could account for the phenotypic differences. Plausibly, MUC2 is of utmost importance, as the functionality of intestinal mucus was proposed to rely primarily on MUC2 encoded mucins [10]. Therefore, the full understanding of the functional divergence and prognostic implications of chicken mucins compared to their mammalian orthologues necessitates identification and comparisons of the gene sequences across species.

Although identification of new MUC family members is ongoing, sequencing of most *MUCIN* genes is hampered due to the highly complex PTS cassettes clustered throughout the gene, and several gaps still remain in mouse and human family members [4], [9], [10]. In the case of the secretory mucins, this can largely be accounted for by the large, frequently repetitive PTS region. The presence of several different polymorphic elements in many of the *MUCIN* genes hinders annotation efforts at the gene and protein levels, and could even hamper the understanding of the biological significance and disease associations of the diverse family members. By using overlapping RT-PCR, long-range PCR and RACE techniques we have cloned an 11,359 bp chicken *MUC2* cDNA. Previous annotations and predictive modeling validate our predicted gene structure. The cDNA that we cloned spans at least 64 exons on chicken 5q16. The central PTS region of the chicken *MUC2* locus harbors four CysD motifs and contains 10 repeat cassettes. Although we have closed the gap across the PTS domain by sequencing overlapping cDNA clones derived primarily from chicken intestinal mRNA, it is likely that future studies using more sophisticated sequencing platforms will identify additional exons within the PTS domain. The highly complex nature of this motif indicates that obtaining the full-length *MUC2* cDNA could be difficult in the absence of single molecule sequencing efforts. This problem is a common occurrence in the delineation of other mucin genes in mouse and human [4], [9], [10].

The 5′-end of the *MUC2* mRNA contains two in-frame ATG codons. Comparing the surrounding sequences of the first ATG codon to the Kozak consensus sequence [42] indicates that the purine at −3 and the G at +6 of GCCGCCATGGGG are conserved within the optimal context for initiation of translation [43]. The sequences surrounding the second ATG codon (Met^10^; GCCTTTTTATGCTC) are non-consensus Kozak sequences with a T at position −3 and a C at +6. Additionally, analysis of human and mouse MUC2 proteins indicates that the first three amino acids are MGL, which strongly indicates that the first in-frame ATG codon is most likely the translational start site.

The initiating methionine residue is followed by a signal sequence of 18 amino acid residues (analyzed by Signal P3.0; HMM probability: 0.997) (http://www.cbs.dtu.dk/services/SignalP-3.0/) [44] that are rich in leucine but not isoleucine, and are plausibly cleaved to generate the mature mucin isoform during mucin biosynthesis. The amino-terminal region of MUC2, from its initiating methionine to the third C8 motif, spans 1,166 residues composed of multiple VWD and two TIL domains. TIL domains consist of 10 cysteines that are capable of forming disulfide bonds, indicating a high degree of secondary and tertiary structure is possible for these heterogeneous MUC2 protein isoforms. The carboxyl-terminus contains a terminal cystine knot (CT), as well as VWC, VWD domains. These domains are highly conserved throughout evolution [3].

In the endoplasmic reticulum, MUC2 forms disulfide-linked dimers via the VWD domains of the amino-terminus [7], [45], while the CT knot in the carboxy-terminus supports disulfide-linked trimerization in the trans-Golgi network [8]. CysD (C8) domains exert non-covalent cross-linkages in the MUC2 gel formation process, likely contributing to tertiary structure and determination of the pore size of the mucus network [12]. Chicken may plausibly carry more CysD domains than that of human, which may suggest that the polymeric net-like structure contains smaller pores in chickens than humans. This could account for differences in innate defense response to pathogens. The conservation of a cationic domain at the C-terminus observed in rodents was not found in chickens [46].

In human MUC2, two different PTS domains have been identified, both of which are located on the same large exon separated by ∼600 bp. One region consists of repeats that are interrupted in places by 21 to 24 bp segments. The other is composed of an uninterrupted array (of up to 115 repeated units) of a tandem 23-amino acid repeat cassette [18]. Due to the highly unpredictable but repetitive nature, the PTS regions are somewhat refractory to traditional cloning and sequencing technologies [24], [47]. In mice, partial cDNA sequences from the PTS domain suggest the presence of two repetitive PTS regions containing 8 or 10 repetitive units interspaced by a cysteine-rich domain [24]. These repeats are highly dissimilar from both the human and chicken PTS domain. The cDNA that we cloned is composed of 10 interspersed segmental duplications, with the following consensus sequence: VSSSSAPPTPTGSSPTTTSGTTP SSSTIGSTVSTTPVTSPPSPSPTSVSTSTPGPTPTTSVTRPPTSTE. The repetitive unit is rich in threonine (30%), proline (22%) and serine (29%), and is especially high in serine compared to human MUC2 (0% per repetitive unit in the human PTS-region 2). The significance of this is not clear, however, the PTS domains are highly modified posttranslationally by oligosaccharides in humans [30], and these differences could play a role in species-specific innate immune response.

The spatio-temporal expression of *MUC2* transcripts follows a specific pattern in humans and rodents [24], [48], [49], [50]. Similarly, our data show that chicken intestinal *MUC2* transcripts are expressed throughout the gastrointestinal tract and in embryos as early as E14.5. This is thereafter followed by a rapid increase that follows a developmental timeline. This pattern is seemingly disrupted during the developmental switch from E21.5 (hatch day) to post-hatch day 1 in the duodenal and jejunal tissue. These types of temporal trends in *MUC2* expression patterns have been linked to a previous morphometric investigation of intestinal goblet cells, where a gradient increase in goblet cell density was observed along the gastrointestinal tract, and during the period from 3 d prior to and 7 d post hatch [51].

## Conclusions

In summary, we have characterized the chicken *MUC2* cDNA and identified several conserved structural features of the chicken gene, including VWC, VWD, TIL, C8 and CT domains, as well as large PTS tandem repeat region. Interestingly, although the VWC, VWD, TIL, C8 and CT domains are highly conserved amongst human, mouse and chicken, the PTS domain is quite divergent. Since MUC2 is highly glycosylated posttranslationally, this diversity could prove to be a valuable method for generating species-specific innate immune responses to different host pathogens. This is supported by the supposition that the different species could create mucin gel layers with vastly different pore sizes. This could hamper the ability of pathogens to invade different species and provide a mechanism for the different responses seen across species. Interestingly, known sequence variations in other species have elicited functional differences in cancer incidence, induction of virulence from pathogens, bacterial mucolysis, amongst others, suggesting that the heterogeneity of MUC2 plays an important role in many different biological processes. By defining the structure of mucin from an avian species, we provide important information pertaining to a deeper understanding of the evolutionary mechanisms by which genes contribute to innate barrier functions in the host amongst a wide variety of species. By understanding the role of MUC2 in innate host defense in chickens, we may be able to develop more effective therapies for creating enhanced defense mechanisms in humans.

## Supporting Information

Figure S1 **Chicken MUC2 cDNA.** We identified an 11,359 bp cDNA for MUC2. We derived the cDNA from all available sources, including: *in silico* data, mRNAs, ESTs, RACE products and RT-PCR amplicons.
(DOCX)

Figure S2 **Chicken MUC2 protein.** Based on the cDNA sequence from Figure S1, we deduced that the MUC2 protein was 3697 amino acids. We used InterProScan (REF) to determine the different domains, and then compared this analysis to the protein structure provided (medkem). When necessary, we adjusted domains to corresponded with the medkem analysis. We used RADAR (REF) to identify repetitive elements within the protein. Domains are noted in bold, color-coded text, which corresponds to the color coding in Figure 8. Color coding is as noted: signal peptide (black), VWD domains (medium blue), C8 domains (fuschia), TIL motifs (orange), VWC domains (dark blue), CysD (green), PTS (purple), Cysteine knot (yellow). Shaded text indicates the location of the 69 bp repeat within the PTS motifs.
(DOCX)
