# Supplementary material for: Cloning, Annotation and Developmental Expression of the Chicken Intestinal MUC2 Gene
Source: PLoS One. 2013 Jan 21;8(1):e53781. doi: 10.1371/journal.pone.0053781 (PMC3549977; doi:10.1371/journal.pone.0053781)
Supplement: Figure S1 — Chicken MUC2 cDNA. We identified an 11,359 bp cDNA for MUC2. We derived the cDNA from all available sources, including: in silico data, mRNAs, ESTs, RACE products and RT-PCR amplicons. (DOCX) [file pone.0053781.s001.docx]

1 CACAGTTCAC CCACCTTAGC CGCCATGGGG CTAAGAGCTG CCAGCCTTTT TATGCTCTGG

61 CTGGCTCTTT CCAACACGAG TGAAATAAGA AAAGGAAGGA CAAGAAACCA TGGCCACTAT

121 GTCTGCAGCA CCTGGGGGAA CAACCATTTC AAAACTTTCG ATGGAGATAT CTATCAATTT

181 CCTGGCATGT GCGAGTATAA TTTTGTTTCT GACTGCCAAG ACTCTTACAG GGAGTTCTCT

241 GTCCACATTC AACGTGCTCT GAACAGCAAT AACCACCCTG AGATCCAGTA TATCCTGATA

301 ACAGTCACGG ATTTCACAGT GTACCTCAAG CCCAAACTGG CTGTTGTGGA TGGGCGGATT

361 GTCAAGACAC CCTACTACAG CTCTGGTTTG CTCATTGAGA GCAATGACAT TTATACCAAG

421 GTTTATGCTA AACTAGGCCT AATTCTGATA TGGAATCAGG AAGATGCACT GATGGTGGAG

481 CTGGACAGCA AGTTTGGTAA CCAGACATGT GGTCTCTGTG GGGATTACAA TGGAGTTCCA

541 ATCTACAATG AGTTTATTAG TGGAGTTGCA AGCTACAATT CAATTACATA TGGGAATTTA

601 CAGAAGATCA GCAAACCCAA TGATGAATGT GAAGACCCTG ATGAAACTCG GGCTCTGCCA

661 AGCTGTAATG AGCATGTGAG TAAGCCTAAC TGTGAACTTC GCTTTCCTGG ATGCATGGAG

721 ACATCCTTGC ACTGTGAGAT GTTACGTGAG GAATGTGAGA AGTTGCTGAC CTCCTCTGCA

781 TTTGCTGATT GTCACTCACG CCTTAATCTG GAAATGTACA TCCAAGCCTG CATGCAGGAC

841 AAGTGTGCAT GTAAAGGAAA TGAGGACTCC TTCTGCCTCT GCAGCACCAT CTCCGAGTAT

901 TCTCGCCAGT GTTCACATGC AGGTGGCCGG CCGGGTGAAT GGAGGACAGA GAACTTTTGC

961 TACAAAACAT GTCCTGGCAA CATGGTCTAT AGAGAAAGCA GCTCACCCTG CATGGATACT

1021 TGCTCACACT TGGAAGTCAG CAGCCTTTGT GAGGAACATT ACATGGATGG TTGTTTCTGC

1081 CCCGAAGGGA CTGTGTATGA TGATATCACA GAAAATGGCT GCATACCTGT GAGCCAGTGC

1141 TCCTGCAGAC TCCATGGAAA GGGATATTCA CCTGGAGAAA CCATTACCAA TGAGTGTGAA

1201 GAATGCACCT GTGATTCAGG CAGATGGACA TGCAAAGATT TACCCTGTCC AGGCACATGT

1261 TCAGTAGAAG GAGGTTCCCA TATTAAGACC TTTGATGGGA AGAAATACAC CTTCCATGGA

1321 GACTGTTACT ACGTGTTGGC CAAGAGTGCT GTGAATGATA CTCATGCCCT CCTGGCAGAG

1381 CTGGCTCCTT GTGGCTCCTC AGATGGACAG ACCTGTTTGA AGACTGTTGT GTTGTTAGTA

1441 GATGACAAAA AAAATGTGGT GGTTTTCAGA TCAGATGGCA GTGTGTCACT GAATGAGATG

1501 ACAGTGAATG TGCCTCATGT GTCAGCTAGC TTCTCAGTTT TCAAGCCATC TTCTTACTAC

1561 CTCATTGTAC ATACTTCTTT TGGGCTTCAG CTGCAGATTC AGTTGTCTCC AGTCATGCAG

1621 CTGTTTGTGA CAGCGGATCA GTCAGTCCAG GACATTCAAG GCAAGTGTCC ACTGAAACAG

1681 GAAGCAAAGC CATGCATTTC CACAGCTGTC AAGTCATATA TTCTTTACAT GGGTGGTCTT

1741 TGTGGAAATT TTAATGGGAT GGAAGGTGAT GACTTTAAAA CAACCAATGG ATTGGTAGAA

1801 GCTACAGGAT CTGCCTTTGC TAACACATGG AAAGCTCAGC CCACCTGTGC AGACCAAGCA

1861 GAAAAGCTGG AAGACCCATG CACTCTTAGC ATTGAAAGTG CAAATTATGC TGAGCATTGG

1921 TGTTCTTTGC TGAAAAACTC AGAGGGCCCA TTTGCAAGAT GTCATTCAGT CATTGATCCT

1981 GCAGAATACT ATAAGAGATG TAAATATGAC ACCTGCCTCT GCAAGGACAA TGAGGAGTGC

2041 TTGTGTGCTG CCCTGTCCTC TTATTCAAGA GCCTGTGCTT TCAAAGGGAT TATACTGGGT

2101 GGCTGGAGGC AAAGTGTCTG CTCTGATGAA GTGTCTGCCT GCCCAGGAAA TCAAGTCTTC

2161 CTCTACAATC TTACAATGTG CCAGCAAACT TGTCGTTCCA TTGCTGATGG TGAAAAGTAC

2221 TGCTTGCAGG ACTTTGCTCC TGTGGATGGC TGTGGCTGCC CAGATAATAC ATACTTGGAT

2281 AATCAGGATA CGTGTGTGCC CATCTCCAAA TGCCCATGCT ATTACAAGGG GTCATATCTG

2341 GAACCTGGGG AATATGTTAC AAAAGATGGA GAACGTTGTG TTTGCCGAAA TGCAAAGATC

2401 CGGTGTACTT CAGTGACAAT GAGAATGAAA AGTATAACAG AATGTCCCTC AAACAAGACC

2461 TACTTTGATT GCAACGCGTC CCCAGACTGG ACTTCACGGA CACCTGTACA ACTTCGCTGT

2521 GGTACTTCTC AAACTGATCT TTATCAAGGT GAATGTGTCT CAGGCTGTGT GTGTCCTGAA

2581 GGTCTGTTTG ATGATGGTAG AGGGGGCTGT GTTGAGGAAG AGGACTGCCC ATGTATTCAT

2641 AACAGTGACT GGTATAACCA TGGACAGAGT ATAACAGTGG ACTGCAACAC CTGTACCTGC

2701 CAGAAAGGTA TCTGGAGCTG CACTAAAAAT GTGTGCTATG GAACTTGCAT GATATATGGA

2761 AGTGGCCATT ATATCACCTT TGATGGAAAA TTCTATGACT TTGATGGAAG TTGTGAATAC

2821 GTGGCTACTC AGGATTATTG CGGTGACAAA AATTCTGGTG GCTCATTCAG TATAATCACA

2881 GAGAACGTCC CTTGTGGAAC TACAGGAGTC ACCTGCTCAA AGGCCATTAA AATGTTTATA

2941 GGGAAAACTG AACTGAAGTT GGAGAATAAG GATTATAAAG AAATTCAGCG AGATGTTGGC

3001 GATGATGTGC ATTACCAGAA CAAGACAGTA GGTCTCTACC TCGTTATTGA AGCCAGCAAT

3061 GGTGTGATGC TTATCTGGGA TAAGAAGACC ACTGTTTTCA TCAAACTGAC TCCTGACTAT

3121 AAAGGGAAAG TGTGTGGTCT GTGTGGCAAC TTTGATGACA AGGCCAACAA TGACTTTACA

3181 AGCAGGAATG GGCTGCAAGA GACAAATGCT TTGACGTTTG GGAATTCCTG GAAACAATCA

3241 TCTGTGTGCC CTGATGTCAC AGAAGAGATT AAGCCCTGTG ATCTGAAACC ACATCGCAAG

3301 TCCTGGGCAG AGAAAGAATG CAGCCTTATC CTGAGTGAAA TCTTTAAAAT TTGTCATTCC

3361 AAGGTGAACC CATCTCCTTT CTATGATGCT TGTGTTCATG ATGCCTGCTC TTGTGACAGT

3421 GGTGGAGACT GTGATTGCTT CTGTTCTGCT GTTGCTGCAT ATGCACAAGA ATGTACCAAG

3481 GCTCAGGCCT GTGTTTTCTG GAGAAGTCCT GATATATGCC CAATATTCTG TGACTACTAT

3541 AACCCTCGTA ATGAGTGTGA ATGGCACTAC GAGCCTTGTG GCAGCAATGT CATGACCTGC

3601 AGAATGCTTT ATAATGTCAG CACCAACTTC TCAGTTCCGT ACCTGGAAGG TTGCTACCCC

3661 AGATGCCCTC ATGAAAAGCC CGTTTATAAT GAAGAGACAA AAGAATGTGT GGCTGCAGAT

3721 GAATGTTGGT GTTACCACAA TGGTAAACCT GTTATCATTG GTGGAGAAAT ACCAACAGAA

3781 GAGAACTGTA CAAAATGTAT CTGTCGCCCC TCAGGATCCA TTGAGTGTAC ACCAATTCCA

3841 GGATGTCCTT GTGTTATCAA TGGAACAAGT TATGAAGTGG GACAAACTGT TGCAACAATA

3901 AAGGATGGCG ACATATGCAC AACATATGTT TGTGCAGAAA ATGGATCTGT AGTTCCTGGA

3961 AGTACTTATC CTTGTCCATT AACTACTTCA CCTTCAACCA TTACAACGTC TCCTGGTACA

4021 AGTACTCTTA CCACCATAGT TACCACAAGT CCTCCAGTAC CTACAACTCC ATGCCTTGTA

4081 TTAATCTGTG ATTGGTCTGA GTGGTTTGAT GTTAGTAACC CTGAAGAAGA TGGTGGTGAC

4141 TATGAAACCT ATGATGCAAT AAGAATTGAA CATGGAAACA AAATATGTGC AGCTCCTGAA

4201 AATATTGAAT GCAGGGCTAA AGACAGTCCT GATAAAACCT TAGAGGAACT TGGCCAAAAA

4261 GTGGAGTGTA ATGTGACGTA TGGGCTCATC TGTAAAAATG AGGAGCAAGA TGCAAGTATC

4321 TGGCATCTTT GCTATAATTA TGAAATCAGG GTAAACTGTT GTGAATGGCA TGAAATTCCT

4381 TGTGACGCTG GACCAAGCTC AACACCAACT GCATCTTCGG TCACCACTGG AACAACTGTG

4441 CCCACTACTA CAACCCAGCA GTCAACGACA ATTACCACCA TACCAACACC CACTGCAACC

4501 ACCACAAGCC AGTCTCCACC TTCAGTAACT TCAGGCTCCA CAGTATCAAC CACTGCTCCC

4561 ACACCAACTC CATCAGAGTC TCCAACCAGT ACTACCACCA CAACACCCTT CAGCAGCAGC

4621 TCGCAGGGAG CTACTCCTAC TGTTCCTGGT GTGTCTTCAT CCATTCCAAC AGTTACAAGC

4681 ACAGTCCTAC CACCAATAGT AACTCAAACC CACTCTCCTC CACCTTCGCA ACCAGTAGGA

4741 AATTGTGAAT GCACCTGGAC AGACTGGATC GATTCGAGTC AACCAGATAG ATCTGATGTG

4801 AACAGTGGAG ACTATGAAAC CATTGACAAA ATAAACAACT CCTCCTTTGT ATGCGTGAAA

4861 GCTGAGAATA TTTCATGCAG AGCAAAGGAT TACCCTAACA TTTCCCTTGA AGAATTAGGG

4921 CAGAAGGTTG AATGCAGCGT TAACACAGGG CTTATCTGTA ACAATAAGGA TCAAGAAAAC

4981 AACGGCCATG TTTCATACTG CCATAACTAT GAGATCAACG TCTGCTGCAC ACCAAACAAA

5041 CCAGAGTGTA TTCCAAGTAC GGAACTTGTC ACGACAATCA GCACACCAGT GTCACACTCA

5101 ACCACTACAA CCATAGTTTT CACTTCAACT GTGTCAAGCA CTGCTGCCAC GACAACACCA

5161 ACTGGGTCTG CCCCCACTAC CACCTCTGGC ACGATGCCCT CCAGCAGCAC CATCGGCAGC

5221 ACCGTGAGCA CCACGCCTGT TACCAGCCCG CCCTCACCCA GCCCGACTTC AGTCAGCACA

5281 TCCACACCCA GGCCAACACC CACCACCTCC GTAACACGTC CTCCCACGTC GACAGAAGGT

5341 CCCACTCCTG AGAGCACAAC ACGTACGACT GTGTCAAGTA GCAGTGCCCC GCCAACACCA

5401 ACTGGATCTA GCCCCACTAC CACCTCTGGC ACGACGCCCT CCAGCAGCAC CATCGGCAGC

5461 ACCGTGAGCA CCACGCCTGT TACCAGCCCG CCCTCACCCA GCCCGACTTC AGTCACCACA

5521 TCCACACCCG GGCCAACACC CACCAGCTCT GTAACACCTC CACCAACGGC AACTGAAATA

5581 GTCTGCACCT TCCCTCCGGA GGACTGCATC TGGACTGATT GGATCGATGT GAGTTACCCA

5641 CAGTTTGGCC CAGAGGGTGG TGATGATGAG ACCTTTGAAA CTATCCAGAA TAAGATCCCT

5701 TCATGGAACT GTACGAAGGT TGAGAATGTT TCCTGCAGAG CTGAGAAATT TCCAGACATT

5761 CCTATAGAAG ATCTAGGGCA GAAAGTGGAA TGCGATGTTA ACACAGGACT CATCTGTAAA

5821 AATAAGGACC AGACCACAGG GGGTGTGATA CCAATGCCGG TCTGCCTGAA TTATCAGATC

5881 AAAGTCTGCT GTGCTCCACC ATTAAGTCCA GAGTGCACAA CAGCTGTCAC AACTACGACG

5941 ACAGGAGAAA CCAGCACAAC AGTGTCCCTC TCACCGACAA CAAGCACTCG TTATACTCCG

6001 AGCGTGTCAA GCACGACTTC CCCACCAACA CCAACTGGGT CTGCCCCCAC TACCACCTCT

6061 GGCACAACGC CCTCCAGCAG CACCATCGGC AGCACCGTGA GCACCACACC TGTTACCAGC

6121 CCGCCCTCAC CCAGCCCGAC TTCAGTCAGC ACATCCACAC CCGGGCCAAC ACCTACCACC

6181 TCTGTAACAC GTCCTCCCAC GTCGACAGAA GGTCCCACTC CTGAGAGCAC AACACGTACG

6241 ACTGTGTCAA GTAGCAGTGC CCCGCCAACA CCAACTGGAT CTAGCCCCAC TACCACCTCT

6301 GGCACGACGC CCTCCAGCAG CACCATCGGC AGCACCGTGA GCACCACGCC TGTTACCAGC

6361 CCGCCCTCAC CCAGCCCGAC TTCAGTCAGC ACATCCACAC CCGGGCCAAC ACCCACCACC

6421 TCCGTAACAC GTCCTCCCAC GTCGACAGAA GGTCCCACTT CCCAGAGCAC CACCAGTACA

6481 ACAGTGTCGA GTCCAAGTGT GTCAAGTAGC AGTGCTCCGC CAACACCAAC TGGATCTAGC

6541 CCCACTACCA CCTCTGGCAC GACGCCCTCC AGCAGCACCA TCGGCAGCAC CGTGAGCACC

6601 ACGCCTGTTA CCAGCCCGCC CTCACCCAGC CCGACTTCAG TCAGCACATC CACACCCGGG

6661 CCAACACCCA CCACCTCCGT AACACGTCCT CCCACGTCGA CAGAAGGTCC CACTCCTGAG

6721 AGCACAACAC GTACGACTGT GTCAAGTAGC AGTGCCCCGC CAACACCAAC TGGATCTAGC

6781 CCCACTACCA CCTCTGGCAC GACGCCCTCC AGCAGCACCA TCGGCAGCAC CGTGAGCACC

6841 ACGCCTGTTA CCAGCCCGCC CTCACCCAGC CCGACTTCAG TCAGCACATC CACACCCGGG

6901 CCAACACCCA CCACCTCCGT AACACGTCCT CCCACGTCGA CAGAAGGTCC CACTTCCCAG

6961 AGCACCACCA GTACAACAGT GTCGAGTCCA AGTGTGTCAA GTAGCAGTGC CCCGCCAACA

7021 CCAACTGGAT CTAGCCCCAC TACCACCTCT GGCACGACGC CCTCCAGCAG CACCATCGGC

7081 AGCACCGTGA GCACCACGCC TGTTACCAGC CCGCCCTCAC CCAGCCCGAC TTCAGTCAGC

7141 ACATCCACAC CCGGGCCAAC ACCCACCACC TCCGTAACAC GTCCTCCCAC GTCGACAGAA

7201 GGTCCCACTC CTGAGAGCAC AACACGTACG ACTGTGTCAA GTAGCAGTGC CCCGCCAACA

7261 CCAACTGGAT CTAGCCCCAC TACCACCTCT GGCACGACGC CCTCCAGCAG CACCATCGGC

7321 AGCACCGTGA GCACCACGCC TGTTACCAGC CCGCCCTCAC CCAGCCCGAC TTCAGTCAGC

7381 ACATCCACAC CCGGGCCAAC ACCCACCAGC TCTGTAACAC CTCCACCAAC GGCAACTGAA

7441 ATAGTCTGCA CCTTCCCTCC GGAGGACTGC ATCTGGACTG ATTGGATCGA TGTGAGTTAC

7501 CCATGGTTTG GCCCAGAGGG TGGTGATGAT GAGACCTTTG AAACTATCCA GAATAAGATC

7561 CCTTCATGGA ACTGTACGAA GGTTGAGAAT GTTTCCTGCA GAGCTGAGAA ATTTCCAGAC

7621 ATTCCTATAG AAGATCTAGG GCAGAAAGTG GAATGCGATG TTAACACAGG ACTCATCTGT

7681 AAAAATAAGG ACCAGACCAC AGGGGGTGTG ATACCAATGC CGGTCTGCCT GAATTATCAG

7741 ATCAAAGTCT GCTGTGCTCC ACCATTAAGT CCAGAGTGCA CAACAGCTGT CACAACTACG

7801 ACGACAGGAG AAACCAGCAC AACAGTGTCC CTCTCACCGA CAACAAGCAC TCGTTATACT

7861 CCGAGCGTGT CAAGCACGAC TTCCCCACCA ACACCAACTG GGTCTGCCCC CACTACCACC

7921 TCTGGCACAA CGCCCTCCAG CAGCACCATC GGCAGCACCG TGAGCACCAC GCCTGTTACC

7981 AGCCCGCCCT CACCCAGCCC GACTTCAGTC AGCACATCCA CACCCGGGCC AACACCTACC

8041 ACCTCTGTAA CACGTCCTCC CACGTCGACA GAAGGTCCCA CTCCTGAGAG CACAACACGT

8101 ACGACTGTGT CAAGTAGCAG TGCCCCGCCA ACGCCAACTG GATCTAGCCC CACTACCACC

8161 TCTGGCACGA CGCCCTCCAG CAGCACCATC GGCAGCACCG TGAGCACCAC GCCTGTTACC

8221 AGCCCGCCCT CACCCAGCCC GACTTCAGTC AGCACATCCA CACCCGGGCC AACACCTACC

8281 ACCTCCGTAA CACGTCCTCC CACGTCGACA GAAGGTAATT GTCTGGCCGT GGTGCCACGT

8341 GTTCCCCTGT TGAGGGAGAA CTTAACTTCT TCGTACAGCA CTACTATTCT TACAGGTTCT

8401 TTTAGCACCG AAGGTACAAC CACTGTGTCT GAGAGCTCTT CAACAGCTTC AGTTTCTACA

8461 CCTGTTAACA CACAATCTAC AGGAGCATCC TCTCCATTGT TGACTCCATC TGGCAGTATG

8521 TCACCACCTT CAGTTTCAAC AGTCCCATCA ACAGTCACGA CATCTGGAAC AACTCCTACG

8581 AAGCCTTTTA CCACCACTTC AGGAACAACC TCTAGTTCTT TTAGTACTGT CACTGCAACC

8641 ACCATTACCA CCTCGGAAAT TGTTTCCTCT GGTTCAGCTA GTACTTCTGG GACAACTCCT

8701 GCATCAAGTA CAGTCACTGT TTCAGGAAGC TCTACATATA GCACATTGAC TACACTCAGT

8761 ACTTTAAGTT CAACTGCTAG TTCCAGCTCT AATTGTACAA TTTCACCTAA TGAAACTCAT

8821 GCGCCGGGTG AGTCATGGTG GCTGTGTAAC TGCACCAAGG CTATATGTGT AGAAAACAAT

8881 ACTGTTGTGA TCGTTCCAGT AATCTGTGAG CCACCTCCTA AACCCACCTG CTCCAATGGG

8941 CTTGCTCCTG TGCAAGTCAT TGATGATGAT CTATGCTGTT GGCATTGGGA GTGTGATTGC

9001 TACTGCACTG GCTGGGGAGA CCCACATTAT ATGACGTTTG ATGGACTGTA CTACAGCTAT

9061 CAAGGGAATT GCACATATGT TCTTGTGGAA GAGATTGAAA AAAGAGTTGA CAACTTTGGT

9121 GTCTACATTG ATAACTACCA TTGTGATGCA CGGGATATAG TTTCCTGTCC ACGAGCACTC

9181 ATTGTGAGAC ATGAGACTCA GGAAGTGCGC ATTGTAACAG TGAAACCAAA TACCCTGGAA

9241 GTAGAGGTGA CTGTTAACAA ACAGCCTGTG GCTTTGCCTT ATAAGAAATT TGGCTTGAGC

9301 GTCTATGAGT CAGGCATAAA TCGTGTAGTG GAAATTCCTG AACTTAAAAT GAATGTGTCC

9361 TTCAATGGCT TGTCATTTTC TATCAGAATG CCCTACAGCC TGTTTGGAAA CAACACTCAA

9421 GGACAGTGTG GCATTTGCAA TAACAACACG GCAGATGACT GCAGGTTGCC AAATGGAAAT

9481 ATTGCAGAAA ACTGTGAAAC TATGGCAGAT TACTGGCAAG TTGTTGATCC CTCCAAACCA

9541 CAGTGTTCTC CAGGTCTAGT TCCTACAAAA GCACCTAGCA CAACGACAGG GCAGCCTTGC

9601 AAAGAATCTT CCCTCTGTGA GCTTCTTTGG GGAAGTGTGT TTGAGAAGTG CCGTGAAGTT

9661 GTTAAGCCTG ATAAGTACTA TGCAGCTTGC GTTTTTGATA GTTGTACACT TCCCGACTTA

9721 GACCTGGAAT GCTCAAGTCT GCAGATCTAT GCAGCTATCT GTGCCGATCA AAATGTCTGC

9781 GTTGACTGGA GAAGTCATAC CAATGGTGTT TGCTGCTCTT ATGAATGTCC CAAACATAAA

9841 GAATATAGAG CATGTGGTCC TATTCAAGAG ACAACCTGCA AGTCAAGTCC ACAAAATGAA

9901 ACTTCAATTA AACAAATTGA AGGCTGTTTC TGTCCTAATG GTACAATGCT GTTCGACTCT

9961 GGTGTGGATG TCTGTGTAAA TACCTGTGGC TGTGTTGGAT TGGATATGAT ACCAAGAGAG

10021 TTTGGAGAAA AGTTCACAGC AGACTGTCAG GACTGTGTTT GCCTGGAAGG TGGAAATGGC

10081 ATTGTATGTG AACATCACAA ATGTCCTGAA CAAAATAAGA AGAGTTGTTC TGGGAAAGGC

10141 TTCTATGAAG TCACTGTAGT CAACTCTGAA GATCCCTGCT GCCCCACTGT CACTTGCAAA

10201 TGCAACACAA GTTTGTGCAC AACTGAACCC CCCAAGTGTA CACTGGGATT TGAAGTTTTT

10261 TCTTATATTC CCAGTGATGA GTGCTGTCCT GTGTACAAGT GTGTTCCCAA GAAGGTTTGT

10321 GTACATCAGA ATGCTGAGTT CTTGCCTAAT TCCTCAGTCT TTGTTGATAA GTGTCACAAT

10381 TGCTTCTGCA CTAATGAAGT TAACATCAGC ACTCAACTGA ATGTCATCTC CTGTGAACGT

10441 ATTCCATGCA ACACATATTG TGAACCAGGA TATGAACTGC AACACGTACA AGGTGAATGT

10501 TGTGGAAAAT GTGTGCAGAC AAAGTGTGTT ATACATACAT CACACAGTTC CAATCTCATA

10561 CTGAATCCTG GAGAGTTTAT AAATGATCCT TACAACAACT GCACCATTTA CAGTTGTACA

10621 AGTATCAAGA ACCAGCTTAT TTCTTCCACA TCTGAAATTA CATGTCCTGC ATTCAACGAG

10681 GAGAGCTGCA AACCTGGAAC TGTTACATTC TTACCTAATG GTTGTTGCAA AACCTGTATA

10741 CCTCTGGATA GCCCCACACC GTGCTCTGTC CGTGAGAGAA AAGACTTCAT TGTCTATAAG

10801 AACTGCCGTT CTCTGGAGAG AGTTGTCCTG ACTGAATGTG AAGGAACATG TGGAACTTTC

10861 TCACTGTACT CTGTTGAGGC CAGTTCTATG GAGCACAGTT GTTCCTGCTG CAAGGAAGTA

10921 GAAACTAGCA TGAAGGAAGT GGAACTGAAA TGTCCCTCTG GGCACTCAAT TACACATAAG

10981 TATGTGTATG TGGAAAGCTG CGGCTGTCAG GACACTCAGT GCATAGTCCC AGAATCTAGT

11041 GAGTCTCAGA GCACAGAAGA AAATGATGAG AGCACTCAAA ATCACAAGAG AAGAGCCATC

11101 AGCTTAACAT CAAAATGAAG CAGAAAAAAG CTAACAGCAC TCAAGTAAAG GAGCTATGCA

11161 CCACTCTGAT CTTCCTTGAC AGCTTTTGAA CTTAGCTTTT CCCCGCTACA GTATCCTGTT

11221 TGTTCTCTAA TTATTTTTAA CAATGCTCTA TTTATTTGTG TACAAAAGTA CAGTGAACTT

11281 TCTTCTGAGT TGCATACACC TATGGATCAT TTTATAATCA ACTTATTTTC TCTGAATGAT

11341 TAAAAGCACA TTAAAAAAA
