# Supplementary material for: Cloning, Annotation and Developmental Expression of the Chicken Intestinal MUC2 Gene
Source: PLoS One. 2013 Jan 21;8(1):e53781. doi: 10.1371/journal.pone.0053781 (PMC3549977; doi:10.1371/journal.pone.0053781)
Supplement: Figure S2 — Chicken MUC2 protein. Based on the cDNA sequence from Figure S1, we deduced that the MUC2 protein was 3697 amino acids. We used InterProScan (REF) to determine the different domains, and then compared this analysis to the protein structure provided (medkem). When necessary, we adjusted domains to corresponded with the medkem analysis. We used RADAR (REF) to identify repetitive elements within the protein. Domains are noted in bold, color-coded text, which corresponds to the color coding in Figure 8. Color coding is as noted: signal peptide (black), VWD domains (medium blue), C8 domains (fuschia), TIL motifs (orange), VWC domains (dark blue), CysD (green), PTS (purple), Cysteine knot (yellow). Shaded text indicates the location of the 69 bp repeat within the PTS motifs. (DOCX) [file pone.0053781.s002.docx]

1 **MGLRAASLFM LWLALSNT**SE IRKGRTRNHG HYV**CSTWGNN HFKTFDGDIY QFPGMCEYNF Signal**

61 **VSDCQDSYRE FSVHIQRALN SNNHPEIQYI LITVTDFTVY LKPKLAVVDG RIVKTPYYSS**

121 **GLLIESNDIY TKVYAKLGLI LIWNQEDALM VELDSKFGNQ TCGLCGDYNG VPIYNEFISG VWD**

181 **VAS**YNSITYG NLQKISKPND ECEDPDETRA LPSCNEHVSK PNCELRFPGC METSLHCEML

241 **REECEKLLTS SAFADCHSRL NLEMYIQACM QDKCACKGNE DSFCLCSTIS EYSRQCSHAG C8**

301 **GRPGEWRTEN** FCYKT**CPGNM** **VYRESSSPCM DTCSHLEVSS LCEEHYMDGC FCPEGTVYDD TIL**

361 **ITENGCIPVS QC**S**CRLHGKG** **YSPGETITNE CEECTCDSGR WTCKDLPCPG TCSVEGGSHI** **VWC**

421 **KTFDGKKYTF HGDCYYVLAK SAVNDTHALL AELAPCGSSD GQTCLKTVVL LVDDKKNVVV VWD**

481 **FRSDGSVSLN EMTVNVPHVS ASFSVFKPSS YYLIVHTSFG LQLQIQLSPV MQLFVTADQS**

541 **VQDIQGKCPL KQEAKPCIST AVKSYILYMG GLCGNFNGME GDDFKTTNG**L VEATGSAFAN

601 TWKAQPTCAD QAEKLED**PCT** **LSIESANYAE HWCSLLKNSE GPFARCHSVI DPAEYYKRCK C8**

661 **YDTCLCKDNE ECLCAALSSY SRACAFKGII** **LGGWRQSVC**S DEVSA**CPGNQ** **VFLYNLTMCQ TIL**

721 **QTCRSIADGE KYCLQDFAPV DGCGCPDNTY LDNQDTCVPI SKC**PCYYKGS YLEPGEYVTK

781 DGERCVCRNA KIRCTSVTMR MKSITECPSN KTYFDCNASP DWTSRTPVQL RCGTSQTDLY

841 QGECVSGCVC PEGLFDDGRG GCVEEEDCPC IHNSDWYNHG QSITVDCNTC TCQKGIWSCT

901 KNVCYGTC**MI** **YGSGHYITFD GKFYDFDGSC EYVATQDYCG DKNSGGSFSI ITENVPCGTT VWD**

961 **GVTCSKAIKM FIGKTELKLE NKDYKEIQRD VGDDVHYQNK TVGLYLVIEA SNGVMLIWDK**

1021 **KTTVFIKLTP DYKGKVCGLC** **GNFDDKANND FTSRNGL**QET NALTFGNSWK QSSVCPDVTE

1081 EIKPCDLKPH RKS**WAEKECS** **LILSEIFKIC HSKVNPSPFY DACVHDACSC DSGGDCDCFC C8**

1141 **SAVAAYAQEC TKAQACVFWR** **SPDICP**IFCD YYNPRNECEW HYEPCGSNVM TCRMLYNVST

1201 NFSVPYLEGC YPRCPHEKPV YNEETKECVA ADECWCYHNG KPVIIGGEIP TEENCTKCIC

1261 RPSGSIECTP IPGCPCVING TSYEVGQTVA TIKDGDICTT YVCAENGSVV PGSTYPCPLT

1321 TSPSTITTSP GTSTLTTIVT TSPPVPTTP**C** **LVLICDWSEW FDVSNPEEDG GDYETYDAIR CysD**

1381 **IEHGNKICAA PENIECRAKD SPDKTLEELG QKVECNVTYG LICKNEEQDA SIWHLCYNYE**

1441 **IRVNCCEWHE IPCDAGPSST PTASSVTTGT TVPTTTTQQS TTITTIPTPT ATTTSQSPPS PTS**

1501 **VTSGSTVSTT APTPTPSESP TSTTTTTPFS SSSQGATPTV PGVSSSIPTV TSTVLPPIVT**

1561 **QTHSPPPSQP VGNCECTWTD** **WIDSSQPDRS DVNSGDYETI DKINNSSFVC VKAENISCRA CysD**

1621 **KDYPNISLEE LGQKVECSVN TGLICNNKDQ ENNGHVSYCH NYEINVCCTP NKPECIPSTE**

1681 **LVTTISTPVS HSTTTTIVFT STVSSTAATT TPTGSAPTTT SGTMPSSSTI GSTVSTTPVT PTS**

1741 **SPPSPSPTSV STSTPRPTPT TSVTRPPTST EGPTPESTTR TTVSSSSAPP TPTGSSPTTT**

1801 **SGTTPSSSTI GSTVSTTPVT SPPSPSPTSV TTSTPGPTPT SSVTPPPTAT EIVCTFPPED CysD**

1861 **CIWTDWIDVS YPQFGPEGGD DETFETIQNK IPSWNCTKVE NVSCRAEKFP DIPIEDLGQK**

1921 **VECDVNTGLI CKNKDQTTGG VIPMPVCLNY QIKVCCAPPL SPECTTAVTT** **TTTGETSTTV**

1981 **SLSPTTSTRY TPSVSSTTSP PTPTGSAPTT TSGTTPSSST IGSTVSTTPV TSPPSPSPTS PTS**

2041 **VSTSTPGPTP TTSVTRPPTS TEGPTPESTT RTTVSSSSAP PTPTGSSPTT TSGTTPSSST**

2101 **IGSTVSTTPV TSPPSPSPTS VSTSTPGPTP TTSVTRPPTS TEGPTSQSTT STTVSSPSVS**

2161 **SSSAPPTPTG SSPTTTSGTT PSSSTIGSTV STTPVTSPPS PSPTSVSTST PGPTPTTSVT**

2221 **RPPTSTEGPT PESTTRTTVS SSSAPPTPTG SSPTTTSGTT PSSSTIGSTV STTPVTSPPS**

2281 **PSPTSVSTST PGPTPTTSVT RPPTSTEGPT SQSTTSTTVS SPSVSSSSAP PTPTGSSPTT**

2341 **TSGTTPSSST IGSTVSTTPV TSPPSPSPTS VSTSTPGPTP TTSVTRPPTS TEGPTPESTT**

2401 **RTTVSSSSAP PTPTGSSPTT TSGTTPSSST IGSTVSTTPV TSPPSPSPTS VSTSTPGPTP**

2461 **TSSVTPPPTA TEIVCTFPPE** **DCIWTDWIDV SYPWFGPEGG DDETFETIQN KIPSWNCTKV CysD**

2521 **ENVSCRAEKF PDIPIEDLGQ KVECDVNTGL ICKNKDQTTG GVIPMPVCLN YQIKVCCAPP**

2581 **LSPECTTAVT** **TTTTGETSTT VSLSPTTSTR YTPSVSSTTS PPTPTGSAPT TTSGTTPSSS PTS**

2641 **TIGSTVSTTP VTSPPSPSPT SVSTSTPGPT PTTSVTRPPT STEGPTPEST TRTTVSSSSA**

2701 **PPTPTGSSPT TTSGTTPSSS TIGSTVSTTP VTSPPSPSPT SVSTSTPGPT PTTSVTRPPT**

2761 **STE**GNCLAVV PRVPLLRENL **TSSYSTTILT** **GSFSTEGTTT VSESSSTASV STPVNTQSTG**

2821 **ASSPLLTPSG SMSPPSVSTV PSTVTTSGTT PTKPFTTTSG TTSSSFSTVT ATTITTSEIV**

2881 **SSGSASTSGT TPASSTVTVS GSSTYSTLTT LSTLSSTASS SS**N**CTISPNE** **THAPGESWWL VWC**

2941 **CNCTKAICVE NNTVVIVPVI CEPPPKPTCS NGLAPVQVID DDLCCWHWEC** DCY**CTGWGDP**

3001 **HYMTFDGLYY SYQGNCTYVL VEEIEKRVDN FGVYIDNYHC DARDIVSCPR ALIVRHETQE VWD**

3061 **VRIVTVKPNT LEVEVTVNKQ PVALPYKKFG LSVYESGINR VVEIPELKMN VSFNGLSFSI**

3121 **RMPYSLFGNN TQGQCGICNN NTADDCRLPN** **G**NIAENCETM ADYWQVVDPS KPQCSPGLVP

3181 TKAPSTTTGQ **PCKESSLCEL LWGSVFEKCR EVVKPDKYYA ACVFDSCTLP DLDLECSSLQ C8**

3241 **IYAAICADQN VCVDWRSHT**N GVCCSYECPK HKEYRACGPI QETTCKSSPQ NETSIKQIEG

3301 CFCPNGTMLF DSGVDVCVNT CGCVGLDMIP REFGEKFTAD **CQDCVCLEGG NGIVCEHHKC VWC**

3361 **PEQNKKSCSG KGFYEVTVVN SEDPCCPTVT C**KCNTSLCTT EPPKCTLGFE VFSYIPSDEC

3421 CPVYKCVPKK VCVHQNAEFL PNSSVFVDKC **HNCFCTNEVN ISTQLNVISC ERIPCNTYCE VWC**

3481 **PGYELQHVQG ECCGKC**VQTK CVIHTSHSSN LILNPGEFIN DPYNNCTIYS CTSIKNQLIS

3541 STSEITCPAF NEESCKPGTV TFLPNG**CCKT** **CIPLDSPTPC SVRERKDFIV YKNCRSLERV CT**

3601 **VLTECEGTCG TFSLYSVEAS SMEHSCSCCK EVETSMKEVE LKCPSGHSIT HKYVYVESCG**

3661 **CQDTQCIVPE SSESQS**TEEN DESTQNHKRR AISLTSK
